# Elucidation of the role of nucleolin as a cell surface receptor for nucleic acid-based adjuvants

**DOI:** 10.1038/s41541-022-00541-6

**Published:** 2022-10-06

**Authors:** Satoki Kitagawa, Teppei Matsuda, Ayaka Washizaki, Hirotomo Murakami, Takuya Yamamoto, Yasuo Yoshioka

**Affiliations:** 1 Laboratory of Nano-Design for Innovative Drug Development, Graduate School of Pharmaceutical Sciences, Osaka University, 1-6 Yamadaoka, Suita, Osaka, 565-0871 Japan; 2 Vaccine Creation Group, BIKEN Innovative Vaccine Research Alliance Laboratories, Research Institute for Microbial Diseases, Osaka University, 3-1 Yamadaoka, Suita, Osaka, 565-0871 Japan; 3 Laboratory of Immunosenescence, National Institutes of Biomedical Innovation, Health and Nutrition, 7-6-8 Saito-Asagi, Ibaraki, Osaka, 567-0085 Japan; 4 Vaccine Creation Group, BIKEN Innovative Vaccine Research Alliance Laboratories, Institute for Open and Transdisciplinary Research Initiatives, Osaka University, 3-1 Yamadaoka, Suita, Osaka, 565-0871 Japan; 5 The Research Foundation for Microbial Diseases of Osaka University, 3-1 Yamadaoka, Suita, Osaka, 565-0871 Japan; 6 Center for Infectious Disease Education and Research, Osaka University, 2-8 Yamadaoka, Suita, Osaka, 565-0871 Japan; 7 Global Center for Medical Engineering and Informatics, Osaka University, 3-1 Yamadaoka, Suita, Osaka, 565-0871 Japan

**Keywords:** Adjuvants, Toll-like receptors

## Abstract

Nucleic acid-based adjuvants such as CpG oligonucleotides (CpG ODNs) and poly(I:C) are potential vaccine adjuvants for infectious diseases and cancers. However, the mechanism by which their cell surface receptors promote their uptake into dendritic cells (DCs) and shuttle them to intracellular Toll-like receptors remains to be further investigated. Here, we demonstrated a role for nucleolin, a multifunctional DNA- and RNA-binding protein and a major constituent of the nucleolus, as one of the cell-surface receptors for nucleic acid-based adjuvants. Nucleolin on mouse DC surface bound directly to A-type CpG ODN, B-type CpG ODN, and poly(I:C) and promoted their internalization into cells following DC maturation in vitro. In human DCs, nucleolin also contributed to the binding and internalization of both types of CpG ODNs and subsequent cytokine production. Furthermore, nucleolin played a crucial role in cytokine production and activating antigen-specific antibodies and T cell responses induced by B-type CpG ODN in vivo in mice. Our findings provide valuable information that can help improve the efficacy and safety of these adjuvants.

## Introduction

The recent COVID-19 pandemic has demonstrated that pathogen-induced infectious diseases pose severe public health threats worldwide. Vaccination is the most critical and reliable way to prevent severe illness and control the spread of pathogens at the population level. Vaccines using inactivated pathogens or pathogen-derived proteins as antigens induce adaptive immune responses, such as pathogen-specific antibodies and T cell responses. In addition, one means of improving the vaccine-induced adaptive immune responses and enhancing protective effects against pathogens is the rational use of the immunomodulatory agents called adjuvants^1^. Currently, a wide variety of adjuvants are being studied in preclinical and clinical trials. For example, aluminum salts are one of the most reliable adjuvants, and many vaccines for infectious diseases formulated with aluminum salts have been used worldwide^1,2^. However, aluminum salts cannot induce antigen-specific Th1-type immune responses and CD8^+^ T cell responses, but can strongly induce antigen-specific antibody responses^1,2^. It is desirable to develop adjuvants to induce these T cell responses as pathogen-specific Th1-type immune responses and cytotoxic CD8^+^ T cell responses are important for the elimination of pathogens, especially viruses^3,4^.

Many nucleic acid-based adjuvants have been developed to efficiently induce Th1-type immune responses and CD8^+^ T cell responses^1,5^. For example, oligodeoxynucleotides (ODNs) with unmethylated cytosine-phosphate-guanine (CpG) motifs (CpG ODNs) are short single-stranded synthetic DNA fragments containing immunostimulatory CpG motifs^6,7^. CpG ODN binds to Toll-like receptor 9 (TLR9) in endosomes upon uptake by antigen-presenting cells (APCs) such as dendritic cells (DCs), B cells, and macrophages^6,7^. There are several types of CpG ODNs, such as A-type CpG ODNs and B-type CpG ODNs, each of which has a different structure and physical properties^6,7^. In addition, among A-type CpG ODNs and B-type CpG ODNs, various types with different nucleic acid sequences have been developed^6,7^. A-type CpG ODNs predominantly activate plasmacytoid DCs (pDCs) to produce type I interferons (IFNs) such as IFN-α^6,7^. By contrast, B-type CpG ODNs activate the production of interleukin (IL)-6 and IL-12 by APCs, but only weakly induce IFN-α production^6,7^. A-type CpG ODNs have a naturally occurring phosphodiester backbone and self-assemble into multimers, such as G-quadruplex-containing structures, because of their poly G sequences^6,7^. B-type CpG ODNs are the most promising adjuvants among nucleic acid-based adjuvants, with a non-natural phosphorothioate backbone with resistance to nuclease degradation^6,7^. A specific B-type CpG ODN has been used in human vaccines against hepatitis B virus^8^. The other type of nucleic acid-based adjuvant is polyinosinic-polycytidylic acid (poly(I:C)). Poly(I:C) is a synthetic analog of double-stranded RNA that binds to TLR3 in endosomes after uptake by APCs^9,10^. In addition, it activates cytosolic nucleic acid sensor melanoma differentiation-associated protein 5 (MDA5), which causes poly(I:C) toxicity via cytokine storm induction^9,10^. In APCs, the binding of CpG ODNs and poly(I:C) to endosomal TLR9 and TLR3, respectively, as well as to MDA5 in the cytoplasm, activates adaptor proteins such as MyD88 to upregulate the expression of pro-inflammatory cytokines and/or type I IFN genes^7,9^. These nucleic acid-based adjuvants can induce antigen-specific antibodies, and Th1-type and CD8^+^ T cell responses through a strong activation of APCs, when vaccinated with antigens. Therefore, these adjuvants are expected to be useful for formulating vaccines for infectious diseases and cancer.

The endosomal localization of TLR9 and TLR3 necessitates APCs to internalize nucleic acid-based adjuvants into cells and shuttle them to endosomes before being recognized by TLRs^6,7,9,10^. However, the uptake mechanism of extracellular nucleic acids, including CpG ODNs and poly(I:C), into cells remains unclear. In particular, the mode of action of specific cell surface receptors that promote the internalization of extracellular nucleic acid-based adjuvants are largely unidentified. However, there is some evidence to implicate the involvement of cell surface receptors for nucleic acids. Previous reports have shown several types of proteins, such as DEC-205, the receptor for advanced glycation end-products (RAGE), CD14, CD93, and CD206, to be the cell surface receptors for binding to extracellular nucleic acids^11–17^. For example, DEC-205 on the cell surface of mouse and human DCs has been shown to recognize B-type CpG ODNs and facilitate their internalization and subsequent recognition by TLR9 in vitro^12,17^. In addition, DEC-205 contributes to cytokine production and DC activation upon intravenous injection of B-type CpG ODNs in mice^12,17^. Other reports have shown that RAGE on DC surface plays a crucial role in the recognition and internalization of A-type CpG ODNs and B-type CpG ODNs in vitro, as well as cytokine production in the lungs of mice^14,15^. We hypothesized that the cell surface receptors for extracellular nucleic acids have redundant and complementary functions. For example, there might be multiple crucial cell surface receptors for extracellular nucleic acids in a single cell type, and different cell types might use other receptors. Therefore, to develop modified nucleic acid-based adjuvants with efficacy and safety, it is essential to correctly identify the cell surface receptors, which contribute to adaptive immune responses induced by nucleic acid-based adjuvants in vivo.

Nucleolin is a multifunctional RNA-, DNA-, and protein-binding protein ubiquitously expressed in eukaryotic cells^18–20^. Nucleolin is a major constituent of the nucleolus and localizes to the nucleus and cytoplasm^18–20^. Nucleolin plays crucial roles in the maturation of pre-ribosomal RNA, transcription of ribosomal DNA, ribosome maturation, and shuttling of RNA from the nucleus to the cytoplasm by interacting with RNA, DNA, and other proteins^18–20^. Furthermore, nucleolin is also located on the cell surface of various types of cells and plays a critical role in modulating the cell cycle, cell proliferation, and apoptosis^18–20^. In particular, the cell surface expression of nucleolin is markedly enhanced in several cancers and indicative of a poor prognosis^18–20^. Therefore, nucleolin-specific DNA aptamers and antibodies are expected to be used as anti-cancer therapeutics and drug delivery vehicles for cancer cells^18–22^. In fact, AS1411, a 26-base DNA aptamer with high affinity and specificity for nucleolin, is the first clinically tested aptamer for cancer therapy and has been used for the targeting of anti-cancer drugs to cancers^19,22^. In addition, nucleolin on the cell surface acts as a receptor for several types of growth factors, such as hepatocyte growth factor and viruses such as human respiratory syncytial virus, and participates in the translocation of several molecules and viruses from the cell surface into cells^19,23,24^. In cultured cardiomyocytes, a recent study showed that cell-surface nucleolin contributes to the binding and internalization of extracellular mitochondrial DNA and CpG ODNs, as well as nuclear factor-κB (NF*-*κB) activation they induce^25^. These facts collectively led us to hypothesize that nucleolin might act as a cell surface receptor for multiple types of nucleic acid-based adjuvants on APCs.

In this study, we demonstrated the potential of nucleolin as a cell surface receptor for nucleic acid-based adjuvants such as CpG ODNs and poly(I:C) in vitro and in vivo. Importantly, we showed that nucleolin acts as the cell surface receptor on mouse DCs for CpG ODNs and poly(I:C). In human DCs, nucleolin plays a crucial role as a cell surface receptor for CpG ODNs. Furthermore, nucleolin contributes to innate and acquired immunity modulated by B-type CpG ODNs in mice. We believe that our results will improve our understanding of the mechanism of action of adjuvants and lead to the development of novel adjuvants designed to improve vaccine efficacy and safety.

## Results

### Binding of CpG ODNs and poly(I:C) to nucleolin

**Fig. 1:**
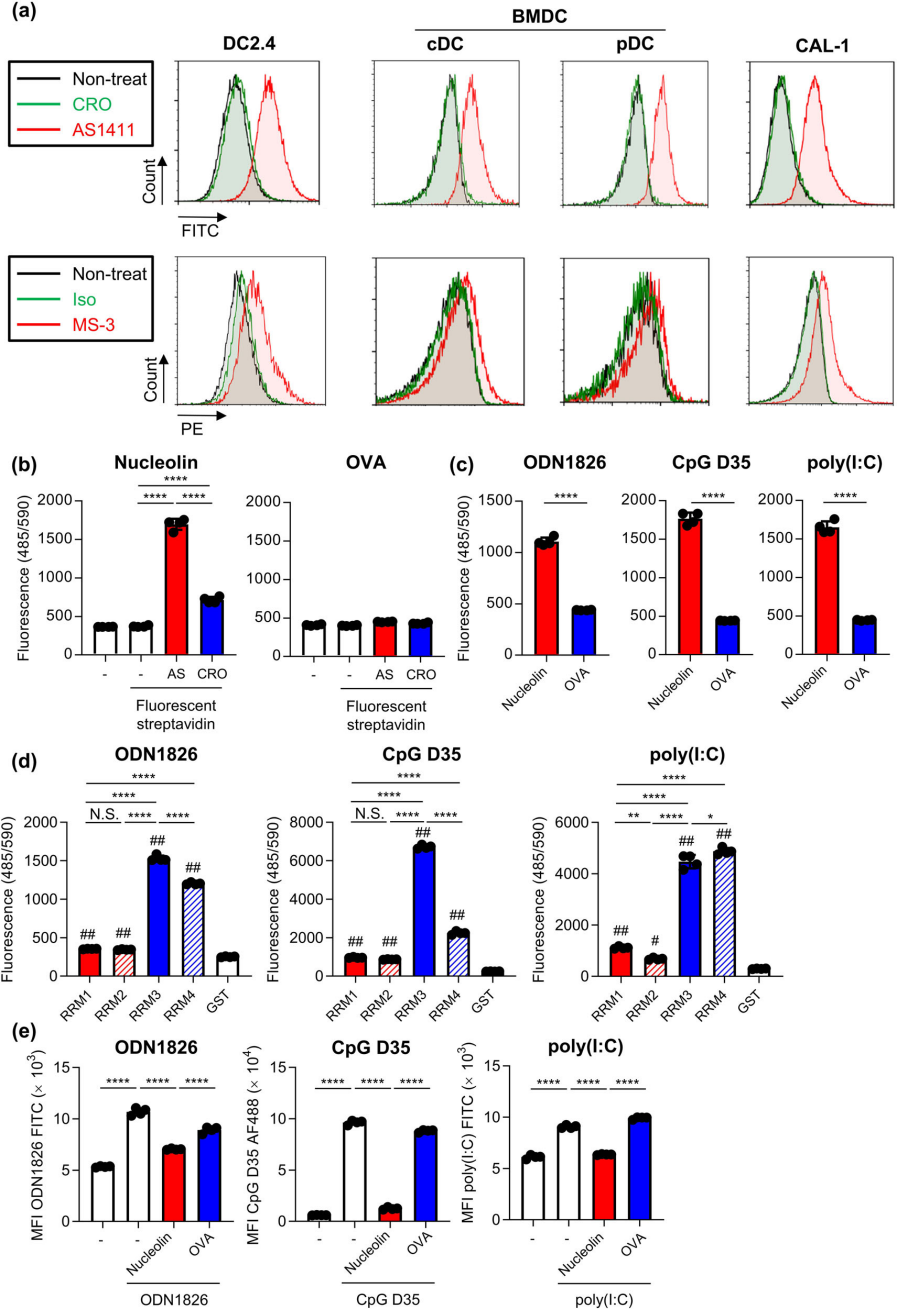
Binding of CpG ODNs and poly(I:C) to nucleolin on the cell surface of DCs. **a** DC2.4 cells, mouse-derived BMDCs, including cDCs and pDCs, and CAL-1 cells were treated with 1 μM FITC-labeled AS1411, 1 μM FITC-labeled CRO, 10 μg/mL PE-labeled MS-3 or 10 μg/mL PE-labeled isotype control antibody at 4 °C for 1 h. The localization of nucleolin on the cell surface was analyzed using flow cytometry. BMDCs were separated into CD11c^+^ PDCA-1^–^ cDCs and CD11c^+^ PDCA-1^+^ pDCs. Binding of **b** biotin-conjugated AS1411 and CRO, **c**, **d** ODN1826, CpG D35, and poly(I:C) to **b**, **c** recombinant nucleolin, OVA, or **d** recombinant RRM, RRM1, RRM2, RRM3, RRM4 measured by using PE-labeled streptavidin. N.S.: not significant. (e) DC2.4 cells were treated with 10 μg/mL FITC-labeled ODN1826, 10 μg/mL Alexa 488-labeled CpG D35, or 10 μg/mL FITC-labeled poly(I:C) with or without 500 μg/mL recombinant nucleolin protein or OVA protein at 37 °C for 30 min. Internalization of FITC-labeled ODN1826, Alexa 488-labeled CpG D35, or FITC-labeled poly(I:C) into DC2.4 cells was measured by flow cytometry after trypan blue quenching. MFI: mean fluorescence intensity. **a**–**e** Each experiment was performed more than twice. **b**–**e** Data are shown as the means ± SD. *P < 0.05, **P < 0.01, ****P < 0.0001 by **b**, **d**, **e** Tukey’s test and **c** Student’s *t*-test. **d** ^#^*P* < 0.01, ^##^*P* < 0.0001 vs. GST group by Tukey’s test.

First, we examined the localization of nucleolin on the cellular surface of DCs using the mouse DC line DC2.4, murine bone-marrow DCs (including conventional DCs (cDCs) and pDCs), and the human DC line CAL-1 (Fig. 1a). We used AS1411, a commonly used DNA aptamer specific to nucleolin, control oligonucleotide (CRO), MS-3, a monoclonal antibody specific to nucleolin, and an isotype control antibody to detect nucleolin. Flow cytometry analysis showed that fluorescent-labeled AS1411 and -MS-3 bound strongly to these DCs at 4 °C, whereas neither CRO nor isotype control antibody bound to these cells (Fig. 1a, Supplementary Fig. 1a). We also confirmed the binding of AS1411 to cDCs, pDCs, B cells, and macrophages from mouse splenocytes at 4 °C (Supplementary Figs. 1b and 2). Next, we examined the expression of nucleolin in the subcellular fractions of CAL-1 cells using western blotting (Supplementary Fig. 3). The purity of the subcellular fractions was confirmed by using antibodies against β-actin for the cytoplasmic fraction, poly(ADP-ribose) polymerase 1 (PARP-1) for the nuclear fraction, and Na^*+*^*/*K^*+*^*-*ATPase for the cell-surface membrane fraction (Supplementary Fig. 3). We observed a 75–100-kDa band for nucleolin in the cytoplasm, nuclear and the membrane fraction (Supplementary Fig. 3). These data suggest that nucleolin is expressed on the cellular surface of mouse and human DCs.

Next, we examined the properties of AS1411 and MS-3 for the following experiments. We observed higher mean fluorescence intensity (MFI) in fluorescent-labeled AS1411- and -MS-3-treated DC2.4 cells at 37 °C than at 4 °C (Supplementary Fig. 4). In addition, quenching of fluorescence on the cell surface by trypan blue treatment more potently decreased MFI at 4 °C than at 37 °C, indicating the possibility of AS1411 and MS-3 being internalized into DCs at 37 °C, but not at 4 °C (Supplementary Fig. 4). We also showed that the binding of fluorescent-labeled MS-3 to DC2.4 cells at 4 °C and the uptake of fluorescent-labeled MS-3 into DC2.4 cells at 37 °C was significantly suppressed by AS1411 compared with CRO (Supplementary Fig. 5). These results indicated that AS1411 and MS-3 recognized similar epitopes on nucleolin.

Nucleolin includes an N-terminal domain; a middle region that contains RNA-recognition motifs (RRMs); and a C-terminal glycine- and arginine-rich (GAR) domain. The N-terminal domain is rich in acidic glutamate/aspartate sequence repeats, which mediate protein-protein interactions^18–20,26^. The middle region contains 4 RRM domains (1 through 4) that mediate interactions with many nucleic acids^18–20,26^. The C-terminal GAR region is also important for nucleolin interactions with nucleic acids and is another protein–protein interaction domain^18–20,26^.

Next, we measured the direct binding of CpG ODNs and poly(I:C) to recombinant nucleolin protein fragments. The largest was nearly full-length; we deleted only the N-terminal region (Supplementary Fig. 6a, b), because nucleolin protein with it is not expressed at high levels in *E. coli*^27^. In addition, we used five other fragments as GST-fusion proteins, RRM1, RRM2, RRM3, RRM4, and RRM; the RRM construct contains all four RRM domains (Supplementary Fig. 6a, c). ODN1826, a B-type, and CpG D35, an A-type, were used as the CpG ODNs. The binding of biotin-conjugated AS1411, CRO, ODN1826, CpG D35, and poly(I:C) to recombinant nucleolin protein was detected using fluorescent-labeled streptavidin (Fig. 1b, c). Biotin-conjugated AS1411 bound to recombinant nucleolin significantly compared with the CRO, whereas AS1411 failed to bind to the negative control protein ovalbumin (OVA) (Fig. 1b). ODN1826, CpG D35, and poly(I:C) also bound to recombinant nucleolin, but did not bind to OVA (Fig. 1c). We also confirmed that MS-3 bound to recombinant nucleolin (Supplementary Fig. 7).

To determine which nucleolin domains are involved in this binding, we found that ODN1826, CpG D35, and poly(I:C) bound to the recombinant nucleolin fragment including all four RRM domains (Supplementary Fig. 8a), indicating that the C-terminal region is not required. Differentiating between the RRM domains, AS1411, ODN1826, CpG D35, and poly(I:C) bound to all 4 RRM domains and exhibited greater affinity to RRM3 and RRM4 than they did to RRM1 and RRM2 (Fig. 1d, Supplementary Fig. 8b). MS-3 also bound to all 4 RRM domains and exhibited more binding to RRM1 and RRM3 than to RRM2 and RRM4 (Supplementary Fig. 8c). To further assess the ability of ODN1826, CpG D35, and poly(I:C) to bind to nucleolin, we examined whether recombinant nucleolin protein and RRM domains inhibit the uptake of fluorescent-labeled ODN1826, CpG D35, and poly(I:C) into DC2.4 cells at 37 °C (Fig. 1e, Supplementary Fig. 9). Flow cytometry analysis revealed that fluorescent-labeled ODN1826, CpG D35, and poly(I:C) were internalized into DC2.4 cells (Fig. 1e). Treatment with recombinant nucleolin protein significantly inhibited their uptake into DC2.4 cells, compared with the control protein OVA treatment (Fig. 1e). In addition, we found that RRM1, RRM3, and RRM4 significantly inhibited the uptake of ODN1826 and poly(I:C) into DC2.4 cells, whereas RRM2 also significantly inhibited the uptake of poly(I:C) into DC2.4 cells (Supplementary Fig. 9). The uptake of CpG D35 was significantly inhibited by RRM1 and RRM3 (Supplementary Fig. 9). These results collectively suggest that ODN1826, CpG D35, and poly(I:C) bind to the nucleolin protein.

### Nucleolin-dependent binding, uptake, and activation of B-type CpG ODNs on mouse DCs

**Fig. 2:**
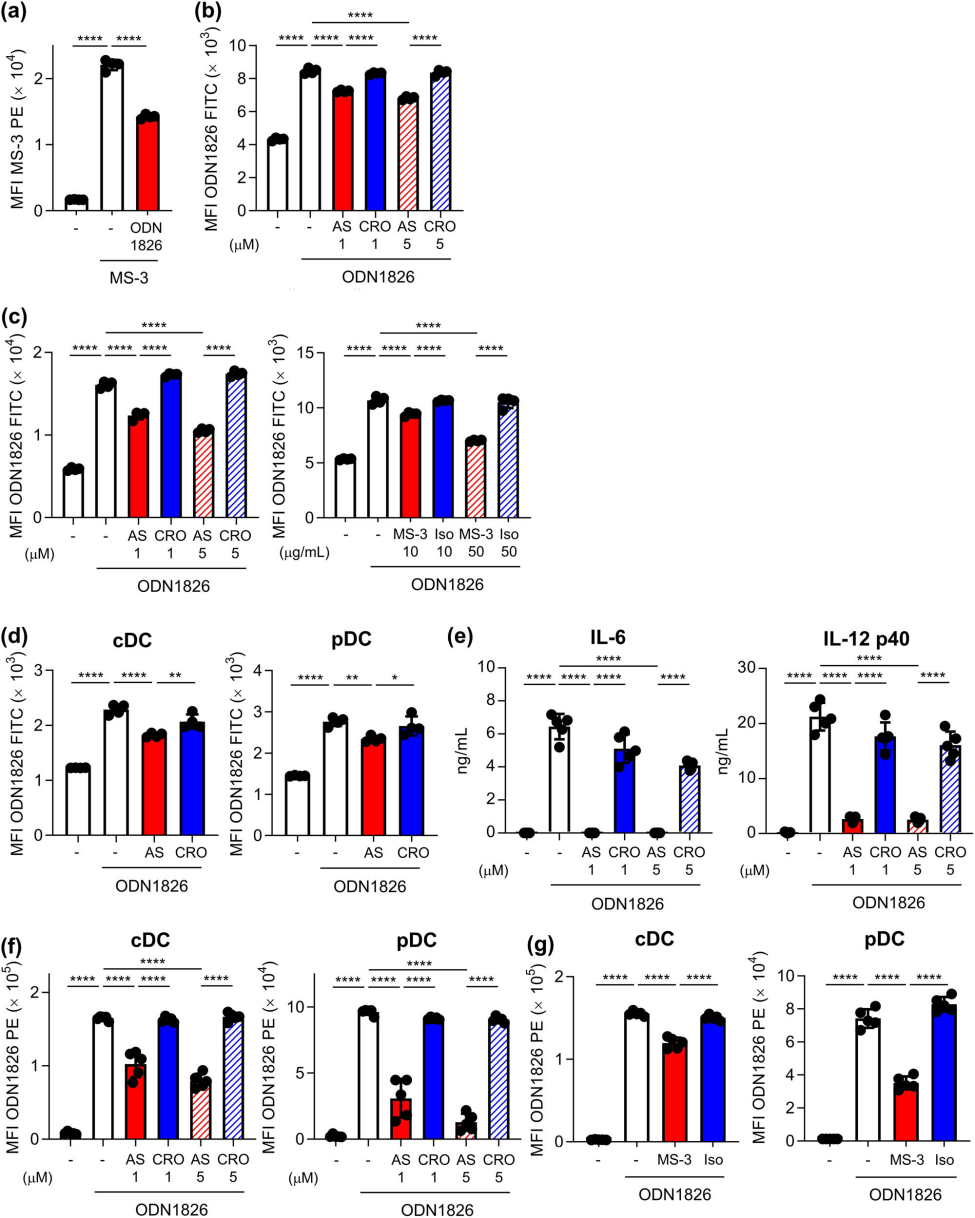
Nucleolin-dependent internalization into DCs and immune responses on DCs of ODN1826, B-type CpG ODN. **a** DC2.4 cells were treated with 10 μg/mL PE-labeled MS-3 with or without ODN1826 at 4 °C for 1 h. The binding of MS-3 on the cell surface of DC2.4 cells was analyzed using flow cytometry. **b** DC2.4 cells were treated with 10 μg/mL FITC-labeled ODN1826 with or without AS1411 (1 or 5 μM) or CRO (1 or 5 μM) at 4 °C for 1 h. The binding of ODN1826 on the cell surface of DC2.4 cells was analyzed using flow cytometry. **c** DC2.4 cells were treated with 10 μg/mL FITC-labeled ODN1826 with or without AS1411 (1 or 5 μM), CRO (1 or 5 μM), MS-3 (10 or 50 μg/mL), or isotype control antibody (10 or 50 μg/mL) at 37 °C for 30 min. Internalization of FITC-labeled ODN1826 into DC2.4 cells was measured by flow cytometry after trypan blue quenching. **d** Mouse-derived BMDCs were treated with 10 μg/mL FITC-labeled ODN1826 with or without 10 μM AS1411 or CRO at 37 °C for 30 min. Internalization of FITC-labeled ODN1826 into BMDCs was measured by flow cytometry. **e**, **f** BMDCs were treated with 0.1 μg/mL ODN1826 with or without AS1411 (1 or 5 μM) or CRO (1 or 5 μM) at 37 °C for 24 h. **e** The concentrations of IL-6 and IL-12 p40 in the supernatants were measured by ELISA. **f** The expression levels of CD86 on cDCs and pDCs were measured using flow cytometry. **g** BMDCs were treated with 0.1 μg/mL ODN1826 with or without 50 μg/mL MS-3 or isotype control antibody at 37 °C for 24 h. The expression levels of CD86 on cDCs and pDCs were measured by flow cytometry. **a**–**g** Each experiment was performed more than twice. Data are shown as the means ± SD. *P < 0.05, **P < 0.01, ****P < 0.0001 by Tukey’s test.

Next, we evaluated the capability of ODN1826 to inhibit the binding of fluorescent-labeled MS-3 to DC2.4 cells at 4 °C using flow cytometry to examine the contribution of the cell surface nucleolin of DCs for the binding of B-type CpG ODNs to DCs (Fig. 2a). ODN1826 significantly suppressed the binding of MS-3 to DC2.4 cells (Fig. 2a). Next, we examined whether AS1411 inhibited the binding of fluorescent-labeled ODN1826 to DC2.4 cells at 4 °C using flow cytometry (Fig. 2b). AS1411 significantly suppressed the binding of fluorescent-labeled ODN1826 to DC2.4 cells compared with CRO (Fig. 2b). In addition, we examined whether AS1411 and MS-3 inhibited the internalization of fluorescent-labeled ODN1826 into DC2.4 cells at 37 °C using flow cytometry (Fig. 2c). Both AS1411 and MS-3 significantly suppressed the uptake of fluorescent-labeled ODN1826 into DC2.4 cells compared with CRO and isotype control antibody (Fig. 2c). We also used mouse-derived BMDCs to test the physiological relevance of nucleolin-dependent ODN1826 uptake into primary DCs (Fig. 2d). Consistent with DC2.4 cells, AS1411 significantly inhibited the uptake of ODN1826 into BMDCs, including cDCs and pDCs, compared with CRO at 37 °C (Fig. 2d). These results collectively suggest that ODN1826 can bind to cell surface nucleolin on mouse DCs, which leads to its subsequent internalization into cells

**Fig. 3:**
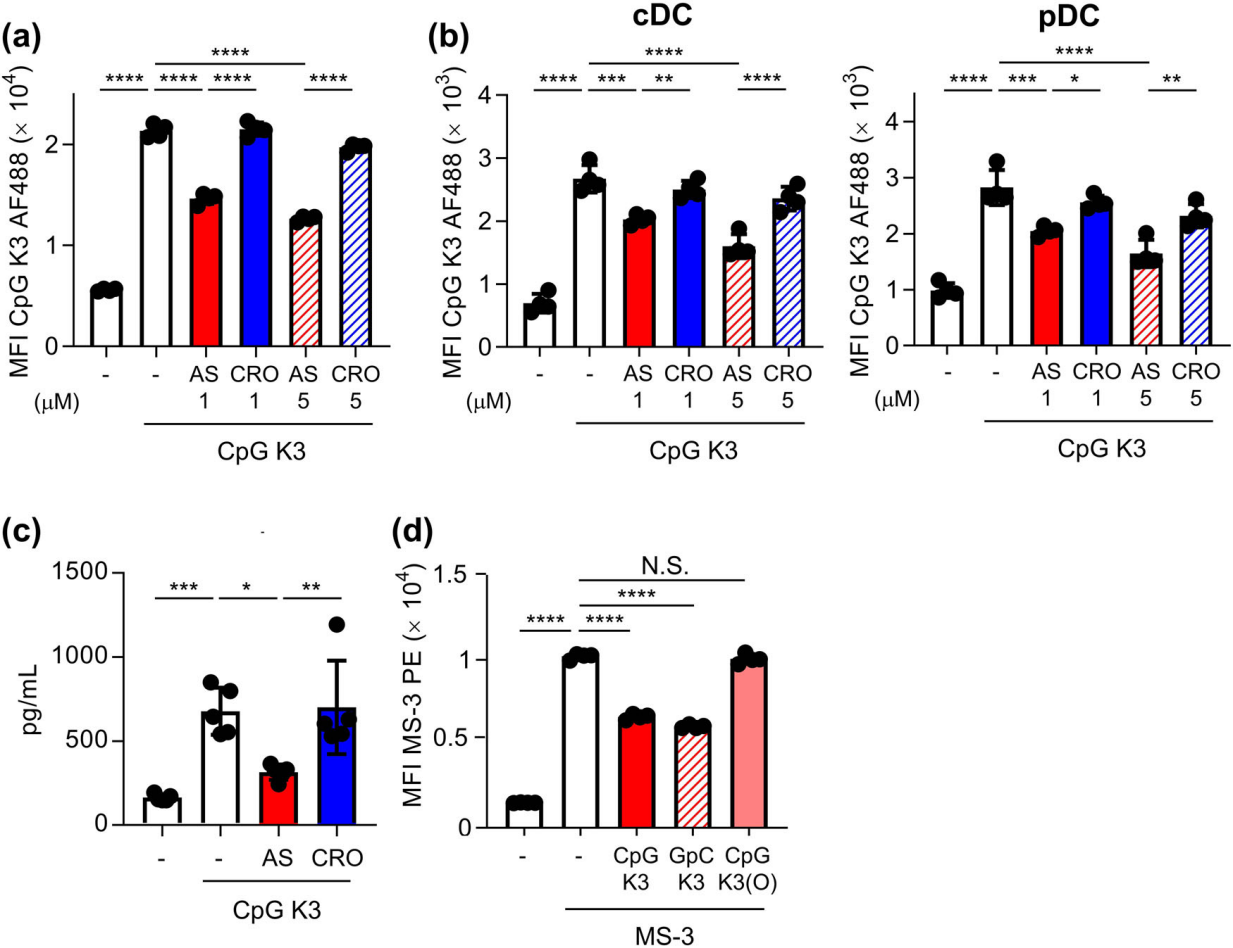
Nucleolin-dependent internalization into DCs and immune responses on DCs of CpG K3, B-type CpG ODN. **a** DC2.4 cells were treated with 10 μg/mL Alexa 488-labeled CpG K3 with or without AS1411 (1 or 5 μM) or CRO (1 or 5 μM) at 37 °C for 30 min. Internalization of Alexa 488-labeled CpG K3 into DC2.4 cells was measured by flow cytometry after trypan blue quenching. **b** Mouse-derived BMDCs were treated with 10 μg/mL Alexa 488-labeled CpG K3 with or without AS1411 (1 or 5 μM) or CRO (1 or 5 μM) at 37 °C for 30 min. Internalization of Alexa 488-labeled CpG K3 into BMDCs was measured by flow cytometry. **c** BMDCs were treated with 1 μg/mL CpG K3 with or without 5 μM AS1411 or CRO at 37 °C for 24 h. The concentration of IL-12 p40 in the supernatants was measured by ELISA. **d** DC2.4 cells were treated with 10 μg/mL PE-labeled MS-3 with or without 50 μg/mL CpG K3, control counterparts of CpG K3 (GpC K3), in which the CpG motif was reversed to GpC, or modified CpG K3 with a phosphodiester backbone (CpG K3(O)) at 4 °C for 1 h. The binding of MS-3 on the cell surface of DC2.4 cells was analyzed by flow cytometry. N.S.: not significant. **a**–**d** Each experiment was performed more than twice. Data are shown as the means ± SD. *P < 0.05, **P < 0.01, ***P < 0.001, ****P < 0.0001 by Tukey’s test.

We examined cytokine production and the expression of co-stimulatory molecules in BMDCs upon treatment with ODN1826 in the presence or absence of AS1411 (Fig. 2e, f). Stimulation of BMDCs with ODN1826 induced massive production of IL-6 and IL-12 p40 (Fig. 2e). IL-6 and IL-12 p40 production induced by ODN1826 was significantly inhibited by treatment with AS1411 compared with CRO (Fig. 2e). We also confirmed that production of IL-12 p70 induced by ODN1826 was significantly inhibited by treatment with AS1411 (Supplementary Fig. 10). Moreover, the enhanced expression of CD86, a representative T cell co-stimulatory molecule, on cDCs and pDCs induced by ODN1826 was also significantly inhibited by AS1411 treatment compared with CRO (Fig. 2f). We also confirmed that the enhanced ODN1826-induced CD86 expression was suppressed by MS-3 treatment compared with the isotype control antibody (Fig. 2g). Next, to verify the versatility of nucleolin for binding to B-type CpG ODNs, we used CpG K3, which is a different type of B-type CpG ODN. The uptake of CpG K3 into DC2.4 cells and BMDCs, including cDCs and pDCs, was significantly inhibited by AS1411 compared with CRO at 37 °C (Fig. 3a, b). IL-12 p40 production from BMDCs induced by CpG K3 was also significantly inhibited by AS1411 compared to CRO (Fig. 3c).

We next examined whether the CpG K3 (GpC K3) control, in which the CpG motif has been reversed to GpC, and CpG K3 modified with a phosphodiester backbone bind to nucleolin (Supplementary Fig. 11a, Fig. 3d). Both CpG K3 and GpC K3 bound to recombinant nucleolin but not to OVA, whereas CpG K3 with a phosphodiester backbone did not bind to nucleolin (Supplementary Fig. 11a). GpC ODN also significantly suppressed the binding of MS-3 to DC2.4 cells at 4 °C (Fig. 3d); in contrast, phosphodiester-modified CpG K3 did not (Fig. 3d). Collectively, these results indicate that nucleolin promotes the uptake of B-type CpG ODNs into mouse DCs via their phosphorothioate backbone and plays a crucial role in mouse DC activation.

To exclude the possibility that AS1411 suppressed the cytokine production independent of nucleolin, we examined whether AS1411 inhibited the cytokine production through cyclic dimeric guanosine monophosphate (c-di-GMP), an agonist for stimulator of IFN genes (STING) that is a cytosolic nucleic acid sensor located in the endoplasmic reticulum^28^. A recent report showed that an immunoreactive cyclic dinucleotide including c-di-GMP is taken up by cells via SLC19A1, a folate-organic phosphate antiporter^29,30^, indicating that nucleolin does not contribute to the uptake of c-di-GMP. We found that c-di-GMP-induced IL-12 p40 production was dependent on STING activation (Supplementary Fig. 12a) and that AS1411 failed to inhibit the induction (Supplementary Fig. 12b). These results suggested that AS1411 did not inhibit the cytokine production non-specifically.

### Nucleolin-dependent binding, uptake, and activation of A-type CpG ODNs on mouse DCs

**Fig. 4:**
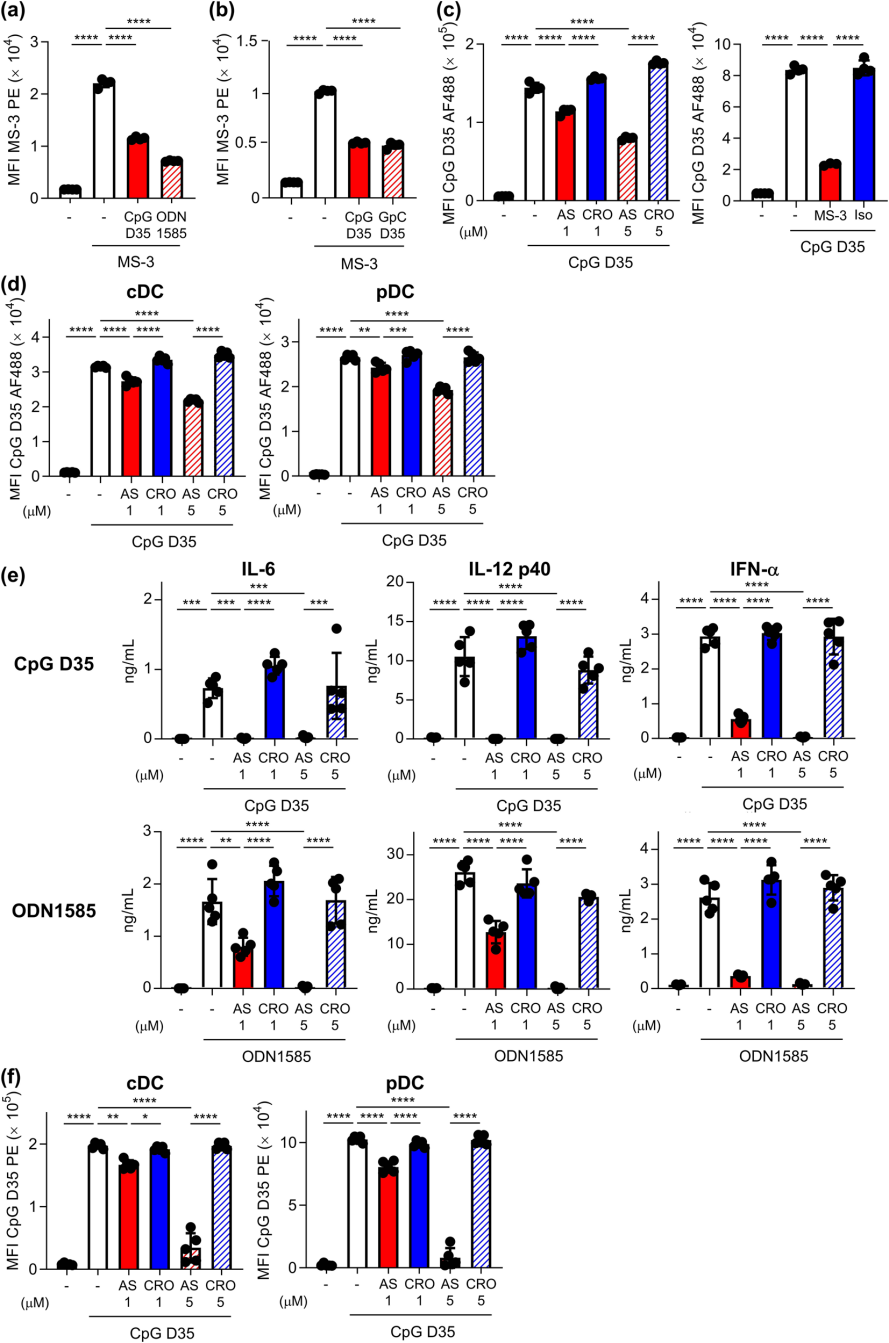
Nucleolin-dependent internalization into DCs and immune responses on DCs of A-type CpG ODNs. DC2.4 cells were treated with 10 μg/mL PE-labeled MS-3 with or without **a** CpG D35 or ODN1585, and **b** CpG D35 or control counterparts of CpG D35 (GpC D35), in which the CpG motif was reversed to GpC, at 4 °C for 1 h. The binding of MS-3 on the cell surface of DC2.4 cells was analyzed by flow cytometry. **c** DC2.4 cells were treated with 10 μg/mL Alexa 488-labeled CpG D35 with or without AS1411 (1 or 5 μM), CRO (1 or 5 μM), 50 μg/mL MS-3, or 50 μg/mL isotype control antibody at 37 °C for 30 min. Internalization of Alexa 488-labeled CpG D35 into DC2.4 cells was measured by flow cytometry after trypan blue quenching. **d** Mouse-derived BMDCs were treated with 10 μg/mL Alexa 488-labeled CpG D35 with or without AS1411 (1 or 5 μM) or CRO (1 or 5 μM) at 37 °C for 30 min. Internalization of Alexa 488-labeled CpG D35 into BMDCs was measured by flow cytometry. **e**, **f** BMDCs were treated with 1 μg/mL CpG D35 or 1 μg/mL ODN1585 with or without AS1411 (1 or 5 μM) or CRO (1 or 5 μM) at 37 °C for 24 h. **e** The concentrations of IL-6, IL-12 p40, and IFN-α in the supernatants were measured by ELISA. **f** The expression levels of CD86 on cDCs and pDCs were measured by flow cytometry. **a**–**f** Each experiment was performed more than twice. Data are shown as the mean ± SD. *P < 0.05, **P < 0.01, ***P < 0.001, ****P < 0.0001 by Tukey’s test.

Next, we examined the contribution of nucleolin to the binding of A-type CpG ODNs to DCs. We used two types of A-type CpG ODNs: CpG D35 and ODN1585. The binding of fluorescent-labeled MS-3 to DC2.4 cells was significantly inhibited by CpG D35 and ODN1585 at 4 °C (Fig. 4a). The counterpart of CpG D35 (GpC D35), in which the CpG motif was reversed to GpC, also significantly suppressed the binding of MS-3 to DC2.4 cells (Fig. 4b). We also confirmed that ODN1585 and GpC D35 bind to recombinant nucleolin protein but not to the OVA control (Supplementary Fig. 11b). In addition, both AS1411 and MS-3 significantly inhibited the uptake of fluorescent-labeled CpG D35 into DC2.4 cells compared with CRO and isotype control antibody at 37 °C (Fig. 4c). We also found that AS1411 significantly inhibited the uptake of CpG D35 into mouse-derived BMDCs, including cDCs and pDCs, compared with CRO at 37 °C (Fig. 4d). CpG D35- and ODN1585-induced IL-6, IL-12 p40, and IFN-α production was significantly inhibited by AS1411 compared with CRO (Fig. 4e, Supplementary Fig. 13). In addition, the enhanced expression of CD86 on cDCs and pDCs induced by CpG D35 was also significantly inhibited by AS1411 compared with CRO (Fig. 4f). These results collectively suggest that A-type CpG ODNs bind to cell surface nucleolin on mouse DCs, which promotes the uptake of A-type CpG ODNs into the cells and plays a crucial role in mouse DC activation.

### Nucleolin-dependent binding, uptake, and activation of poly(I:C) on mouse DCs

**Fig. 5:**
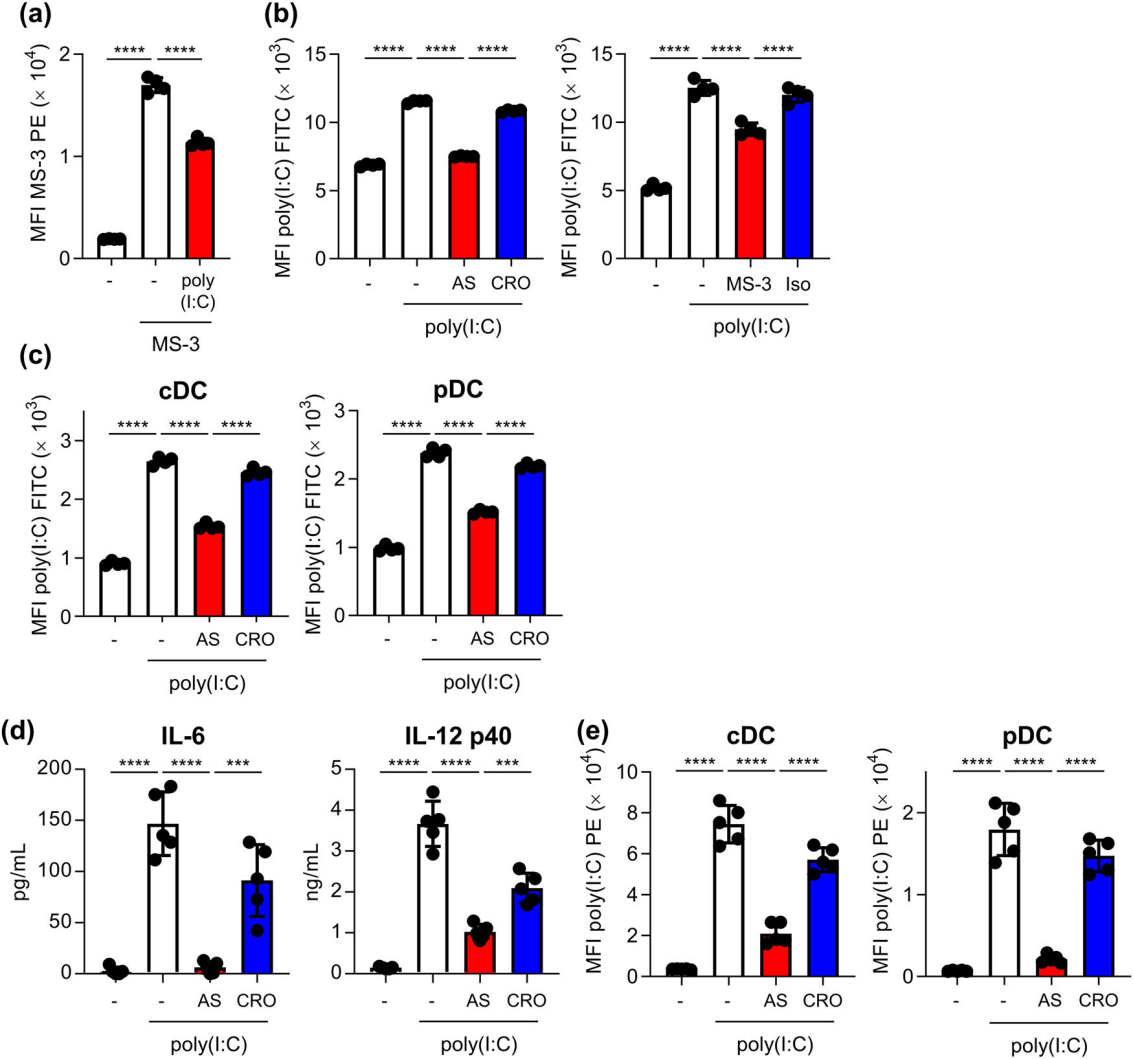
Nucleolin-dependent internalization into DCs and immune responses on DCs of poly(I:C). **a** DC2.4 cells were treated with 10 μg/mL PE-labeled MS-3 with or without 50 μg/mL poly(I:C) at 4 °C for 1 h. The binding of MS-3 on the cell surface of DC2.4 cells was analyzed by flow cytometry. **b** DC2.4 cells were treated with 5 μg/mL FITC-labeled poly(I:C) with or without 5 μM AS1411, 5 μM CRO, 50 μg/mL MS-3, or 50 μg/mL isotype control antibody at 37 °C for 30 min. Internalization of FITC-labeled poly(I:C) into DC2.4 cells was measured by flow cytometry after trypan blue quenching. **c** Mouse-derived BMDCs were treated with 5 μg/mL FITC-labeled poly(I:C) with or without 5 μM AS1411 or 5 μM CRO at 37 °C for 30 min. Internalization of FITC-labeled poly(I:C) into BMDCs was measured by flow cytometry. **d**, **e** BMDCs were treated with 1 μg/mL poly(I:C) with or without 5 μM AS1411 or 5 μM CRO at 37 °C for 24 h. **d** The concentrations of IL-6 and IL-12 p40 in the supernatants were then measured by ELISA. **e** The expression levels of CD86 on cDCs and pDCs were measured by flow cytometry. **a**–**e** Each experiment was performed more than twice. Data are shown as the means ± SD. *P < 0.05, ***P < 0.001, ****P < 0.0001 by Tukey’s test.

We examined the contribution of nucleolin to the binding of poly(I:C) to DCs. Poly(I:C) significantly suppressed the binding of fluorescent-labeled MS-3 to DC2.4 cells at 4 °C (Fig. 5a). AS1411 and MS-3 significantly inhibited the uptake of fluorescent-labeled poly(I:C) into DC2.4 cells, compared with CRO and isotype control antibody at 37 °C (Fig. 5b). In addition, AS1411 significantly inhibited the uptake of poly(I:C) into BMDCs, including cDCs and pDCs, compared with CRO at 37 °C (Fig. 5c). Furthermore, AS1411 significantly inhibited IL-6 and IL-12 p40 production induced by poly(I:C) compared to CRO (Fig. 5d). The poly(I:C)-induced enhanced expression of CD86 on cDCs and pDCs was also significantly inhibited by AS1411 compared with CRO (Fig. 5e). These results suggest that nucleolin contributes to the binding of poly(I:C) to mouse DCs and promotes mouse DC activation by enhancing the internalization of poly(I:C) into mouse DCs.

### Contribution of nucleolin on human DCs

**Fig. 6:**
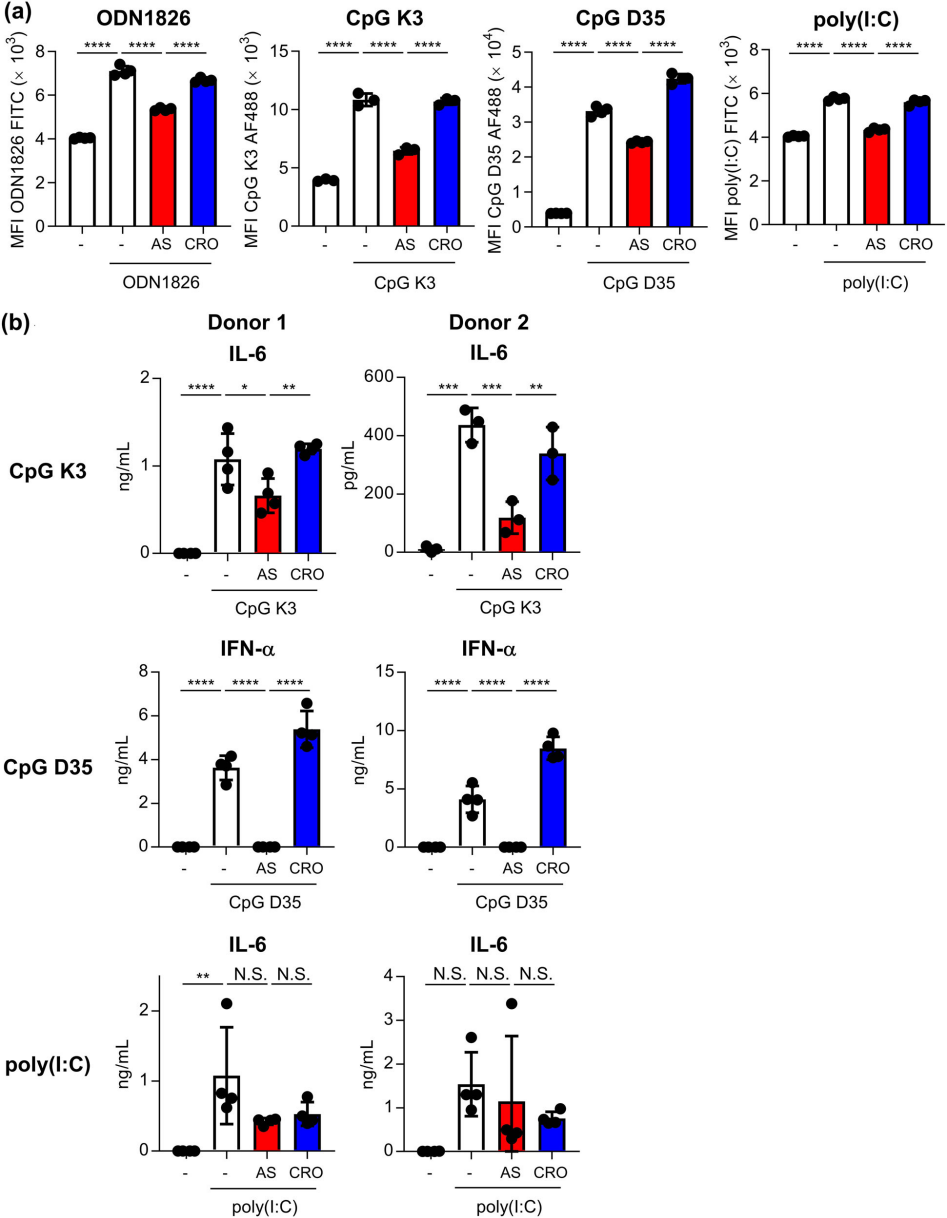
Contribution of nucleolin on CpG ODN- and poly(I:C)-mediated internalization into human DCs and cytokine production from human PBMCs. **a** CAL-1 cells were treated with 10 μg/mL FITC-labeled ODN1826, 10 μg/mL Alexa 488-labeled CpG K3, 10 μg/mL Alexa 488-labeled CpG D35 or 5 μg/mL FITC-labeled poly(I:C) with or without 5 μM AS1411 or 5 μM CRO at 37 °C for 30 min. Internalization into CAL-1 cells was measured by flow cytometry after trypan blue quenching. **b** Human PBMCs were treated with 1 μg/mL CpG K3, 10 μg/mL CpG D35, or 1 μg/mL poly(I:C) with or without AS1411 (1 or 5 μM) or CRO (1 or 5 μM) at 37 °C for 24 h. The concentrations of IL-6 and IFN-α in the supernatants were then measured by ELISA. N.S.: not significant. **a**, **b** Each experiment was performed more than twice. Data are shown as the means ± SD. *P < 0.05, **P < 0.01, ***P < 0.001, ****P < 0.0001 by Tukey’s test.

We then examined the contribution of nucleolin to the binding of CpG ODNs and poly(I:C) to human DCs using human DC line CAL-1 cells. First, we showed that AS1411 significantly inhibited the uptake of fluorescent-labeled ODN1826, CpG K3, CpG D35, and poly(I:C) into CAL-1 cells compared with CRO at 37 °C (Fig. 6a). Next, we examined the contribution of nucleolin to cytokine production induced by CpG ODNs and poly(I:C) from human peripheral blood mononuclear cells (PBMCs) (Fig. 6b). CpG K3-induced production of IL-6 and CpG D35-induced production of IFN-α was significantly inhibited by AS1411 compared with CRO (Fig. 6b). By contrast, AS1411 failed to inhibit both PBMC IL-6 production (Fig. 6b) and the enhanced expression of CD80 on CD14^+^ monocytes (Supplementary Fig. 14) induced by poly(I:C). These results suggest that nucleolin promotes the uptake of CpG ODNs into human DCs following cytokine production. In addition, these results suggest that nucleolin does not contribute to cytokine production by poly(I:C) in human DCs, although it promotes the uptake of poly(I:C) into human DCs.

### Contribution of nucleolin to cytokine production and acquired immunity induced by B-type CpG ODN in vivo

**Fig. 7:**
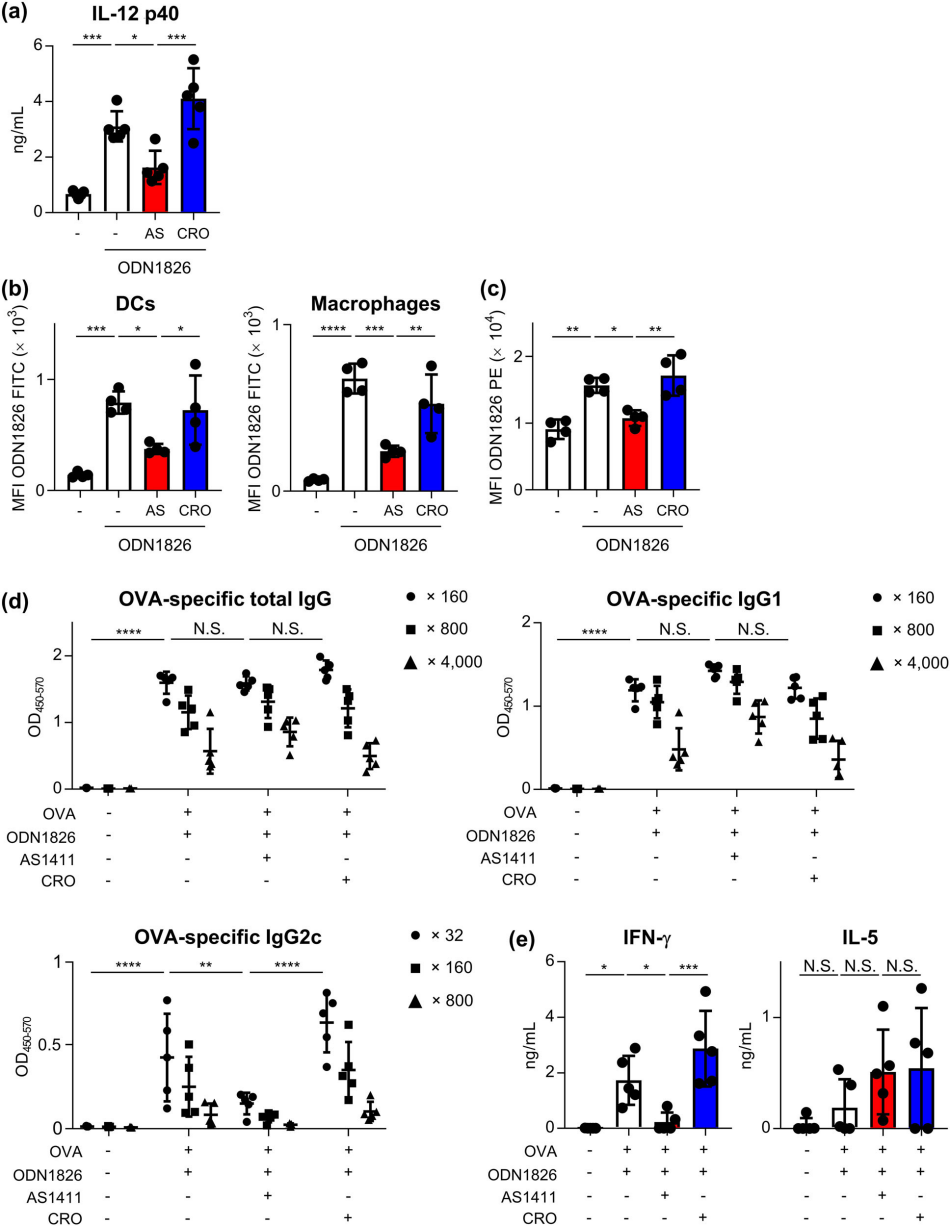
Nucleolin-mediated cytokine production and acquired immunity by B-type CpG ODN in mice. **a** Mice were intravenously administered 1 μg ODN1826, 1 μg ODN1826 plus 4 nmol AS1411, or 1 μg ODN1826 plus 4 nmol CRO. After 3 h, we measured the level of IL-12 p40 in serum by ELISA. **b** Mice were subcutaneously injected with 1 μg FITC-labeled ODN1826, 1 μg FITC-labeled ODN1826 plus 1.5 nmol AS1411, or 1 μg FITC-labeled ODN1826 plus 1.5 nmol CRO. After 30 min, we measured fluorescence of ODN1826 in DCs and macrophages from draining lymph nodes by flow cytometry. **c** Mice were subcutaneously injected with 1 μg ODN1826, 1 μg ODN1826 plus 1.5 nmol AS1411, or 1 μg ODN1826 plus 1.5 nmol CRO. After 24 h, the expression of CD86 on DCs from draining lymph nodes was measured by flow cytometry. **d**, **e** Mice were immunized with 10 μg OVA with 1 μg ODN1826, 1 μg ODN1826 plus 4 nmol AS1411, or 1 μg ODN1826 plus 4 nmol CRO subcutaneously. As a control, mice were treated with medium subcutaneously. **d** After the final immunization, the levels of OVA-specific total IgG, IgG1, and IgG2c in the plasma were measured by ELISA. We used 160- (●), 800- (■), and 4,000- (▲) fold-diluted plasma samples for total IgG and IgG1, and 32- (●), 160- (■), and 800- (▲) fold-diluted plasma samples for IgG2c. N.S. not significant. Significant differences were analyzed only in 160-fold-diluted plasma samples for total IgG and IgG1, and 32-fold-diluted plasma samples for IgG2c. **e** Splenocytes from immunized mice were incubated with OVA in vitro, and IFN-γ and IL-5 in supernatants were quantified. N.S.: not significant. **a**–**e** Each experiment was performed more than twice. Data are shown as the means ± SD. *P < 0.05, **P < 0.01, ***P < 0.001, ****P < 0.0001 by Tukey’s test.

The involvement of nucleolin in B-type CpG ODN-induced immune responses in vivo was evaluated in mice. First, we examined the contribution of nucleolin to innate immune responses induced by the B-type CpG ODN. ODN1826 was injected into mice intravenously with or without AS1411 or CRO, and we measured the concentrations of IL-12 p40 in plasma 3 h after injection (Fig. 7a). The concentrations of IL-12 p40 were significantly higher in the ODN1826-treated mice than in control non-treated mice (Fig. 7a). Furthermore, administration of AS1411 with ODN1826 decreased IL-12 p40 production induced by ODN1826, while CRO did not suppress IL-12 p40 production (Fig. 7a). These data suggest that nucleolin contributes to cytokine production by B-type CpG ODNs in vivo.

Next, we examined the role of nucleolin in the distribution of ODN1826 either with or without AS1411 or CRO injected subcutaneously into mice. Via flow cytometry, we measured the amount of FITC-labeled ODN1826 in DCs and macrophages sampled from draining lymph nodes (Fig. 7b). AS1411 decreased the distribution of FITC-labeled ODN1826 into DCs and macrophages, while CRO had no significant effect (Fig. 7b). Moreover, AS1411 decreased the enhanced expression of CD86 on DCs induced by ODN1826, while CRO had no significant effect (Fig. 7c).

Next, we examined the contribution of nucleolin to the adaptive immune responses induced by ODN1826. Mice were immunized subcutaneously with OVA plus ODN1826, OVA plus ODN1826 and AS1411, or OVA plus ODN1826 and CRO, and the levels of OVA-specific IgG were measured in the plasma after immunization (Fig. 7d). To investigate OVA-specific CD4^+^ T cell responses, splenocytes from immunized mice were stimulated with OVA in vitro, then we measured the secretion of IFN-γ for the Th1 response and IL-5 for the Th2 response (Fig. 7e). Mice immunized with OVA plus ODN1826 showed significantly higher levels of OVA-specific total IgG, IgG1, and IgG2c than control mice (Fig. 7d). Furthermore, the levels of OVA-specific IgG2c in mice immunized with OVA plus ODN1826 and AS1411 were significantly lower than those in mice immunized with OVA plus ODN1826, and OVA plus ODN1826 and CRO, while there was no significant difference in the levels of OVA-specific total IgG and IgG1 among these groups (Fig. 7d). The level of IFN-γ produced from splenocytes in OVA plus ODN1826 and AS1411-immunized mice was also significantly lower than that in OVA plus ODN1826, and OVA plus ODN1826 and CRO (Fig. 7e). In contrast, we did not observe a significant difference in IL-5 production between OVA plus ODN1826 and AS1411-immunized mice and OVA plus ODN1826 and CRO-immunized mice (Fig. 7e). These data suggest that nucleolin contributes to the induction of antigen-specific antibodies and T cell responses by ODN1826 in mice.

## Discussion

Nucleolin is essential for cell survival; previous studies have shown that siRNA-mediated complete knockdown of nucleolin mRNA leads to cell-cycle arrest^31,32^. Therefore, we used AS1411 and MS-3 to block the interaction between nucleic acid-based adjuvants and nucleolin. First, we demonstrated that nucleolin is located in both nucleus and cytoplasm, as well as on the cell surface of mouse-derived DCs, B cells, macrophages, and human-derived DCs using flow cytometry and western blotting. The binding of extracellular ligands to the cell surface nucleolin results in the clustering of nucleolin, leading to the internalization of nucleolin by an actin- and calcium-dependent processes^33,34^. We also showed that binding to cell surface nucleolin lead to efficient internalization of AS1411 and MS-3 as well as CpG ODNs and poly(I:C) into DCs, although we did not examine the dependence on actin and calcium. On the other hand, nucleolin does not have a transmembrane domain and is located on the cell surface via interactions with other cell surface proteins^18^. For example, the binding of nucleolin to Fas on the cell surface blocks the interaction of Fas with Fas ligand, preventing Fas-mediated apoptosis induction^35^. However, it remains unclear which proteins interact with nucleolin on the cell surface of DCs, and further investigation is needed.

MS-3 recognizes an epitope similar to that of AS1411^36,37^; we also found that AS1411 competitively inhibited the binding of MS-3 to DCs, although there remains a possibility that AS1411 induces a conformational change preventing the binding of MS-3 to nucleolin. In this study, we demonstrated that AS1411, CpG ODNs, and poly(I:C) directly bind to recombinant nucleolin via all 4 RRM domains. In addition, our data suggest that RRM3 and RRM4 are especially important for the binding of ODN1826 and poly(I:C), and RRM3 is especially important for the binding of CpG D35. CpG ODNs and poly(I:C) significantly inhibited the binding of MS-3 to the DC surface, while AS1411 and MS-3 also significantly inhibited the uptake of CpG ODNs and poly(I:C) into DCs. These results collectively suggested that CpG ODNs and poly(I:C) might bind to similar, but not exactly the same epitopes, in nucleolin. Moreover, nucleolin might have the potential to interact with several types of nucleic acid-based adjuvants. Future research will necessitate the clarification of the precise binding strength of CpG ODNs and poly(I:C) to nucleolin.

Nucleolin interacts with G-quadruplex-containing DNA structures in nucleus^26^ and AS1411 forms G-quadruplex-containing structures, which contribute to nucleolin binding^19^. Consistent with AS1411, A-type CpG ODNs also form a G-quadruplex-containing structure^6,7^, which indicates that A-type CpG ODNs may bind to nucleolin via such structures. In contrast to A-type CpG ODNs, B-type CpG ODNs do not form G-quadruplex-containing structures and have a phosphorothioate backbone^6,7^. We showed that a B-type CpG ODN with a phosphodiester backbone did not bind to recombinant nucleolin and did not inhibit the binding of MS-3 to DC2.4 cells, while B-type CpG ODN with a phosphorothioate backbone did both. Therefore, a phosphorothioate backbone contributes to nucleolin binding by B-type CpG ODN. We showed that modified ODNs lacking CpG motifs, as well as CpG ODNs, bound to recombinant nucleolin and inhibited the MS-3 binding to DC2.4 cells, indicating that the CpG motif is not necessary for binding to nucleolin, despite its requirement for signaling via TLR9.

Here, we showed that nucleolin increased the sensitivity of mouse DCs to cytokine production and co-stimulatory molecule expression by CpG ODNs and poly(I:C) by promoting their uptake into mouse DCs in vitro. Consistent with the results from mouse DCs, we showed that nucleolin plays a crucial role in the uptake of A-type CpG ODNs and B-type CpG ODNs in human CAL-1 cells, and promoting cytokine production from human PBMCs. By contrast, poly(I:C) did not require nucleolin to induce cytokine production and enhanced expression of co-stimulatory molecule in human PBMCs, although nucleolin contributed to the uptake of poly(I:C) into CAL-1 cells. These results suggested that nucleolin is important for the internalization of poly(I:C) into human DCs, similar to CpG ODNs. In fact, a previous report showed the possibility that poly(I:C) and B-type CpG ODNs might share the same cell surface receptor in human DCs^38^. However, it is not clear as to why nucleolin-mediated uptake of poly(I:C) in human DCs cannot induce DC activation. Further investigation is needed to clarify the intracellular localization of CpG ODNs and poly(I:C) after binding to cell surface nucleolin to identify other cell surface receptors for cytokine production by poly(I:C) in human DCs.

Here, we showed that nucleolin plays a crucial role in cytokine production upon intravenous injection and adaptive immune responses after subcutaneous injection induced by B-type CpG ODN in mice. A previous report showed that cytokine production by B-type CpG ODN after intravenous injection is suppressed in DEC-205 knockout mice compared with wild-type mice^12,17^. Therefore, both DEC-205- and nucleolin-mediated cellular uptake facilitate cytokine production after intravenous injection in mice. In addition, a previous report showed that RAGE contributes to cytokine production in the lungs by B-type CpG ODN in mice^14,15^. It is necessary to examine the contribution of nucleolin to regulate cytokine production by B-type CpG ODNs in the lungs of mice. We showed that nucleolin plays a crucial role in murine antigen-specific antibody and T cell responses, especially Th1 responses, induced by B-type CpG ODN after subcutaneous immunization with antigen plus B-type CpG ODN. These results collectively provide valuable information for designing B-type CpG ODNs as vaccine adjuvants with greater efficacy and safety.

Cell surface receptors for extracellular nucleic acids have redundant and complementary functions, and it is important to investigate the potential of such receptors for nucleic acid-based adjuvants in their function in mouse cells in vitro, their function in human cells in vitro, and their function in vivo. We believe that our results have the potential to improve our understanding of the underlying mechanisms of adjuvant effects and development of novel adjuvants with superior vaccine efficacy and safety.

## Methods

### Reagents

ODN1826 (5′-tccatgacgttcctgacgtt-3′), fluorescein isothiocyanate (FITC)-labeled ODN1826, poly(I:C) HMW, FITC-labeled poly(I:C), ODN1585 (5′-ggggtcaacgttgagggggg-3′), c-di-GMP, and H-151 were purchased from InvivoGen (San Diego, CA, USA). CpG K3 (5′-atcgactctcgagcgttctc-3′), GpC K3 (5′-atgcactctgcaggcttctc-3′), modified CpG K3 with a phosphodiester backbone (CpG K3(O); 5′-atcgactctcgagcgttctc-3′), CpG D35 (5′-ggtgcatcgatgcagggggg-3′), and GpC D35 (5′-ggtgcatgcatgcagggggg-3′) were purchased from GeneDesign (Osaka, Japan). AS1411 (5′-tttggtggtggtggttgtggtggtggtgg-3′), FITC-labeled AS1411, CRO (5′-tttcctcctcctccttctcctcctcctcct-3′) and FITC-labeled CRO were synthesized at Hokkaido System Science (Hokkaido, Japan). Anti-nucleolin monoclonal antibody (clone: MS-3, catalog numbers: sc-8031) and phycoerythrin (PE)-labeled anti-nucleolin monoclonal antibody (clone: MS-3, catalog numbers: sc-8031 PE) were purchased from Santa Cruz Biotechnology (Santa Cruz, CA, USA). Mouse IgG1 isotype control (clone: T8E5, catalog numbers: mabg1-ctrlm) was purchased from InvivoGen. PE-labeled mouse IgG1 isotype control antibody (clone: MOPC-21, catalog numbers: 400112) was purchased from BioLegend (San Diego, CA, USA).

### Cells

DC2.4 cells, a mouse dendritic cell line, was kindly provided by Dr. KL Rock (Department of Pathology, University of Massachusetts Medical School, Worcester, MA, USA). DC2.4 cells were cultured in RPMI1640 supplemented with 10% fetal calf serum, 1% penicillin and streptomycin, 1% nonessential amino acids, 10 mM HEPES, and 2-mercaptoethanol. CAL-1 cells, a human pDC line established from a lymphoma patient, was kindly provided by Dr. Takahiro Maeda (Nagasaki University, Nagasaki, Japan)^39^. CAL-1 cells were cultured in RPMI1640 supplemented with 10% fetal calf serum, 1% penicillin and streptomycin, 1% nonessential amino acids, 10 mM HEPES, and 1 mM sodium pyruvate. DC2.4 cells and CAL-1 cells were maintained at 37 °C in a humidified incubator with 5% CO_2_. To generate murine BMDCs, we isolated bone marrow cells from the femurs of C57BL/6J mice and cultured them at 37 °C for 7 or 9 days with 100 or 300 ng/mL human Fms-related tyrosine kinase 3 ligand (Miltenyi Biotech, Bergisch Gladbach, Germany).

### Mice

C57BL/6J mice (6–7-week-old) were purchased from SLC (Hamamatsu, Japan). Mice were housed in a room with a 12-h:12-h light:dark cycle (lights on, 8:00 a.m.; lights off, 8:00 p.m.) and provided unrestricted access to food and water.

### Expression levels of cell surface nucleolin on DCs

DC2.4 cells, mouse-derived BMDCs, and CAL-1 cells (1 × 10^5^ cells) were incubated with 10 μg/mL PE-labeled MS-3, 10 μg/mL PE-labeled isotype control antibody, 10 μg/mL FITC-labeled AS1411, or 10 μg/mL FITC-labeled CRO at 4 °C for 1 h in 1% bovine serum albumin (BSA) in PBS. The cells were then analyzed by flow cytometry (NovoCyte Flow Cytometer, ACEA Biosciences, San Diego, CA, USA).

### Western blotting analysis

Nuclear or cytoplasmic protein fractions of CAL-1 cells were obtained using NE-PER Nuclear Cytoplasmic Extraction Reagents (Thermo Fisher Scientific, Hampton, NH, USA). The membrane protein fraction of CAL-1 cells was determined using the Mem-PER Plus Membrane Protein Extraction Kit (Thermo Fisher Scientific). For SDS-PAGE, a mixture of purified protein and sample buffer solution (Nacalai Tesque, Kyoto, Japan) containing 2-mercaptoethanol was added to a 10% Mini-PROTEAN TGX Precast Protein Gel (Bio-Rad, Hercules, CA, USA). After gel electrophoresis, proteins were blotted onto polyvinylidene fluoride membranes (Bio-Rad). Immunoblotting was performed in 5% skim milk (w/v) diluted in distilled water. The following primary antibodies were used: mouse monoclonal anti-human nucleolin (clone D-6, catalog numbers: sc-17826, dilution 1/1000; Santa Cruz), mouse monoclonal anti-human β-actin (clone AC-74, catalog numbers: A2228, dilution 1/30,000; Sigma-Aldrich), mouse monoclonal anti-PARP-1 (clone F-2, catalog numbers: sc-8007, dilution 1/1000; Santa Cruz), and rabbit monoclonal anti-Na^*+*^*/*K^*+*^*-*ATPase (clone EP1845Y, catalog numbers: ab76020, dilution 1/10,000; Abcam, Cambridge, MA, USA). Horseradish peroxidase-conjugated goat anti-mouse IgG (catalog numbers: AP503P, dilution 1/5000; Merck Millipore, Darmstadt, Germany) or goat anti-rabbit IgG (catalog numbers: 458, dilution 1/5000; MBL, Tokyo, Japan) were used as secondary antibodies and detected using a ChemiDoc Touch Imaging System (Bio-Rad).

### Recombinant nucleolin expression

A cDNA encoding murine nucleolin (GenBank accession number: X07699.1) corresponding to amino acid residues 286-707, was cloned into the pET11a vector (Merck Millipore). Fragments of this cDNA encoding all four RRM domains, RRM1, RRM2, RRM3, and RRM4 (residues 309-644, 309-385, 395-468, 487-561, and 569-644), respectively were cloned into the pGEX-6P-2 vector (Merck Millipore). Transformed BL21 (DE3) cells were grown at 37 °C to an OD600 of 0.5. Protein expression was induced by adding 0.2 mM isopropyl-β-D-1-thiogalactopyranoside followed by shaking for 4 h at 37 °C. After incubation, bacteria were pelleted by centrifugation at 8000 × *g* for 10 min; the pellet was resuspended in a buffer containing 100 mM NaCl, 5 mM imidazole, and 20 mM Tris-HCl supplemented with protease inhibitors. The bacteria were lysed by sonication. Soluble protein was purified from the cleared cell lysate using an AKTAexplorer chromatography system with a Ni-Sepharose HisTrap FF column (GE Healthcare, Diegem, Belgium) and a Superose 6 Increase 10/300 GL column (GE Healthcare). Recombinant nucleolin was quantified using a Pierce BCA protein assay kit (Thermo Fisher Scientific) with a BSA standard.

### Binding of CpG ODNs or poly(I:C) to recombinant nucleolin fragments

White OptiPlate-96 (Perkin-Elmer, Norwalk, Connecticut, USA) microplates were coated with 10 μg/mL of nucleolin, RRM, RRM1, RRM2, RRM3, RRM4, GST, or OVA in carbonate buffer overnight at 4 °C. The coated plates were washed with PBS containing 0.05% Tween-20 and incubated with PBS containing 1% BSA for 1 h at 25 °C. After washing, 0.2 μM ODN1826, CpG K3, GpC K3, CpG K3(O), CpG D35, ODN1585, GpC D35, CRO or AS1411; or 2 μg/mL poly(I:C), all labeled with biotin, were incubated for 2 h at 25 °C, then with PE-labeled streptavidin (BioLegend) for 1 h at 25 °C. In the other experiment, the plates were incubated with 50 μg/mL PE-labeled MS-3 or PE-labeled isotype control antibody for 2 h at 25 °C. After washing the plates, fluorescence was measured using a multi-plate reader (DS Pharma Biomedical, Osaka, Japan).

### Inhibition of MS-3-binding to DC2.4 cells by CpG ODNs and poly(I:C)

DC2.4 Cells (1 × 10^5^ cells) were incubated with 10 μg/mL PE-labeled MS-3 with or without 50 μg/mL ODN1826, 50 μg/mL CpG K3, 50 μg/mL CpG D35, 50 μg/mL ODN1585, or 50 μg/mL poly(I:C) for 1 h at 4 °C in PBS containing 1% BSA. The cells were then analyzed using flow cytometry.

### Binding and internalization of CpG ODNs or poly(I:C) into DCs

DC2.4 cells, mouse-derived BMDCs, and CAL-1 cells (1 × 10^5^ cells) were incubated with 10 μg/mL FITC-labeled ODN1826, Alexa 488-labeled CpG K3, Alexa 488-labeled CpG D35, or FITC-labeled poly(I:C) with or without 500 μg/mL recombinant nucleolin protein, 500 μg/mL recombinant RRM proteins, 500 μg/mL OVA, AS1411 (1, 5, or 10 μM), CRO (1, 5, or 10 μM), MS-3 (10 or 50 μg/mL), or isotype control antibody (10 or 50 μg/mL) for 1 h at 4 °C or for 30 min at 37 °C in PBS containing 1% BSA. The cells were then analyzed using flow cytometry (NovoCyte Flow Cytometer). For the experiments in DC2.4 cells and CAL-1 cells at 37 °C, cells were stained with 0.08% trypan blue solution (Wako, Osaka, Japan) to quench any fluorescence bound to the cell surface, and then analyzed using flow cytometry.

### Stimulation of mouse-derived BMDCs

Murine BMDCs were seeded at a density of 1 × 10^5^ per well in a 96-well flat-bottomed culture plate. Cells were stimulated with 0.1 μg/mL ODN1826, 1 μg/mL CpG K3, 1 or 10 μg/mL CpG D35, 1 μg/mL ODN1585, 1 μg/mL poly(I:C), or 10 μg/mL c-di-GMP with or without AS1411 (1 or 5 μM), CRO (1 or 5 μM), MS-3 (50 μg/mL), isotype control antibody (50 μg/mL), or 0.4 μg/mL H-151 for 24 h at 37 °C. Supernatants were subjected to ELISA to measure IL-6 (BioLegend), IL-12 p40 (BioLegend), IL-12 p70 (BioLegend), and IFN-α (InvivoGen) secretion in accordance with the manufacturer’s instructions. To check the surface levels of CD86 on DCs, we incubated the cells with anti-mouse CD16/CD32 antibody (clone: 93, catalog numbers: 101302, dilution 1/200; BioLegend), APC-Cy7-labelled anti-CD11c antibody (clone: N418, catalog numbers: 117324, dilution 1/200; BioLegend), APC-labelled anti-PDCA-1 antibody (clone: 927, catalog numbers: 127016, dilution 1/200; BioLegend), and PE-labelled anti-CD86 antibody (clone: GL-1, catalog numbers: 105008, dilution 1/200; BioLegend). DCs were separated into the following subsets: CD11c^+^ PDCA-1^-^ cDCs and CD11c^+^ PDCA-1^+^ pDCs. The cells were then analyzed using flow cytometry.

### Stimulation of human PBMCs

Human PBMCs were obtained from two healthy adult male and female Japanese volunteers who provided informed consent. PBMCs were isolated from whole human blood by centrifugation with Ficoll-Paque at 1,500 rpm for 15 min at 24 °C. PBMCs were cultured in RPMI1640 medium supplemented with 10% fetal calf serum and 1% penicillin and streptomycin. PBMCs were seeded at a density of 5 × 10^5^ cells/well for CpG K3 and poly(I:C) or 2 × 10^5^ cells/well for CpG D35 in a 96-well flat-bottomed culture plate and were cultured in RPMI1640 supplemented with 10% fetal calf serum, 1% penicillin, and streptomycin. These cells were stimulated with 1 μg/mL CpG K3, 10 μg/mL CpG D35, or 1 μg/mL poly(I:C) with or without 5 μM AS1411 or 5 μM CRO for CpG K3 and CpG D35, or with or without 1 μM AS1411 or 1 μM CRO for poly(I:C) for 24 h. Supernatants were subjected to ELISA to determine the levels of IL-6 (BD Bioscience, San Jose, CA, USA) and IFN-α (MABTECH, Nacka Strand, Sweden), according to the manufacturer’s instructions. To measure CD80 expression on CD14^+^ monocytes, 2 × 10^6^ PMBCs/well were stimulated with 1 μg/mL poly(I:C), with or without 1 μM AS1411 or 1 μM CRO, for 12 h. We incubated the cells with LIVE/DEAD™ Fixable Violet Dead Cell Stain Kit (Invitrogen, Waltham, MA, USA), APC-Cy7-labelled anti-CD3 antibody (clone: SP34-2, catalog numbers: 557757, dilution 1/100; BD Biosciences, San Jose, CA, USA), BUV805-labelled anti-CD14 antibody (clone: M5E2, catalog numbers: 565779, dilution 1/400; BD Biosciences), Alexa Fluor 647-labelled anti-CD16 antibody (clone: 3G8, catalog numbers: 557710, dilution 1/200; BD Biosciences), BUV661-labelled anti-CD19 antibody (clone: SJ25C1, catalog numbers: 750536, dilution 1/400; BD Biosciences), Alexa Fluor 700-labelled anti-CD56 antibody (clone: B159, catalog numbers: 561902, dilution 1/400; BD Biosciences), BV750-labelled anti-CD80 antibody (clone: L307.4, catalog numbers: 747001, dilution 1/200; BD Biosciences), and PE-Texas Red-labelled anti-HLR-DR antibody (clone: TU36, catalog numbers: MHLDR17, dilution 1/100; Invitrogen). CD14^+^ monocytes were separated into the subsets: CD3^−^ CD19^−^ CD56^−^ CD16^−^ HLA-DR^+^ CD14^+^ monocytes. Cells were analyzed using flow cytometry.

### ODN distribution and cytokine production in mice

C57BL/6 J mice were injected intravenously with ODN1826 (1 μg/mouse) with or without AS1411 (4 nmol/mouse) or CRO (4 nmol/mouse). Three hours later, we obtained serum samples and measured the concentrations of IL-12 p40 in plasma using an ELISA kit (BioLegend) in accordance with the manufacturer’s instructions. To measure the distribution of ODN1826 on DCs, mice were injected subcutaneously with FITC-labeled ODN1826 (1 μg/mouse) with or without AS1411 (1.5 nmol/mouse) or CRO (1.5 nmol/mouse). Mice were euthanized after 30 min and draining lymph nodes were excised. To measure expression of CD86 on DCs, mice were injected subcutaneously with ODN1826 (1 μg/mouse) with or without AS1411 (1.5 nmol/mouse) or CRO (1.5 nmol/mouse). Mice were euthanized after 24 h and draining lymph nodes were excised. Nodes were incubated with 200 μg/mL Liberase TL (Roche Diagnostics GmbH, Mannheim, Germany) and 10 U/mL DNase I (Roche Diagnostics GmbH) for 60 min at 37 °C. Cells were incubated with anti-mouse CD16/CD32 antibody (clone: 93, catalog numbers: 101302, dilution 1/200; BioLegend), AF700-labelled anti-CD19 antibody (clone: 6D5, catalog numbers: 115528, dilution 1/200; BioLegend), PerCP/Cy5.5-labelled anti-CD11c antibody (clone: N418, catalog numbers: 117328, dilution 1/200; BioLegend), PE/Cy7-labelled anti-CD11b antibody (clone: M1/70, catalog numbers: 101216, dilution 1/200; BioLegend), and PE-labelled anti-CD86 antibody (clone: GL-1, catalog numbers: 105008, dilution 1/200; BioLegend). DCs and macrophages were separated into the subsets: CD19^−^ CD11c^+^ DCs and CD19^−^ CD11c^−^ CD11b^+^ macrophages. Cells were analyzed using flow cytometry.

### Vaccination of mice

OVA with low endotoxin content (Fujifilm, Tokyo, Japan) was used for immunization. Grade V OVA (Sigma-Aldrich) was used for ELISA. C57BL/6J mice were immunized with OVA (10 μg/mouse) and ODN1826 (1 μg/mouse) in the presence or absence of AS1411 (4 nmol/mouse) or CRO (4 nmol/mouse) subcutaneously at the base of the tail on days 0 and 21. On day 28 or 29, we obtained plasma samples, and the levels of OVA-specific antibodies in the plasma were determined by ELISA. ELISA plates were coated with OVA (10 μg/mL) in carbonate buffer overnight at 4 °C to detect OVA-specific IgG, IgG1, and IgG2c. The coated plates were then incubated with 1% Block Ace (DS Pharma Biomedical) for 2 h at 25 °C. Plasma samples were diluted with 0.4% Block Ace, and the dilutions were added to the antigen-coated plates. After incubation with plasma for 2 h at 25 °C, the coated plates were incubated with a horseradish peroxidase-conjugated goat anti-mouse IgG (catalog numbers: AP503P, dilution 1/5000; Merck Millipore), IgG1 (catalog numbers: 1070-05, dilution 1/8000; SouthernBiotech, Birmingham, AL, USA), or IgG2c (catalog numbers: 1079-05, dilution 1/8000; SouthernBiotech) solution for 1 h at 25 °C. After incubation, the color reaction was developed with 0.8 mM tetramethyl benzidine (Dojindo, Kumamoto, Japan) in 100 mM citric acid buffer, stopped with 2 N H_2_SO_4_, and measured at OD450−570 on a microplate reader (Power Wave HT, BioTek, Winooski, VT, USA). On day 28, spleens were collected, and splenocytes were prepared to determine IFN-γ production. Splenocytes (1 × 10^6^ cells) were added to the wells of a 96-well plate and stimulated with 100 μg/mL OVA for 3 or 5 days at 37 °C. After incubation, the concentrations of IFN-γ and IL-5 in the supernatants were measured using an ELISA kit (BioLegend) according to the manufacturer’s instructions.

### Statistical analyses

Statistical analyses were performed using the Prism software (GraphPad Software, San Diego, CA, USA). All data are presented as the mean ± standard deviation (SD). Significant differences were determined using the Student’s *t*-test or Tukey’s test.

### Reporting summary

Further information on research design is available in the Nature Research Reporting Summary linked to this article.

## Supplementary information

Supplemental Figure

REPORTING SUMMARY

## Data Availability

The data supporting the findings of this study are presented in the article and supplementary material. Further information and requests for resources and reagents should be directed to and fulfilled by the lead contact Yasuo Yoshioka (y-yoshioka@biken.osaka-u.ac.jp).
