# Supplementary material for: Elucidation of the role of nucleolin as a cell surface receptor for nucleic acid-based adjuvants
Source: NPJ Vaccines. 2022 Oct 6;7:115. doi: 10.1038/s41541-022-00541-6 (PMC9537314; doi:10.1038/s41541-022-00541-6)
Supplement: Supplementary file 1 — Supplemental Figure [file 41541_2022_541_MOESM1_ESM.pdf]

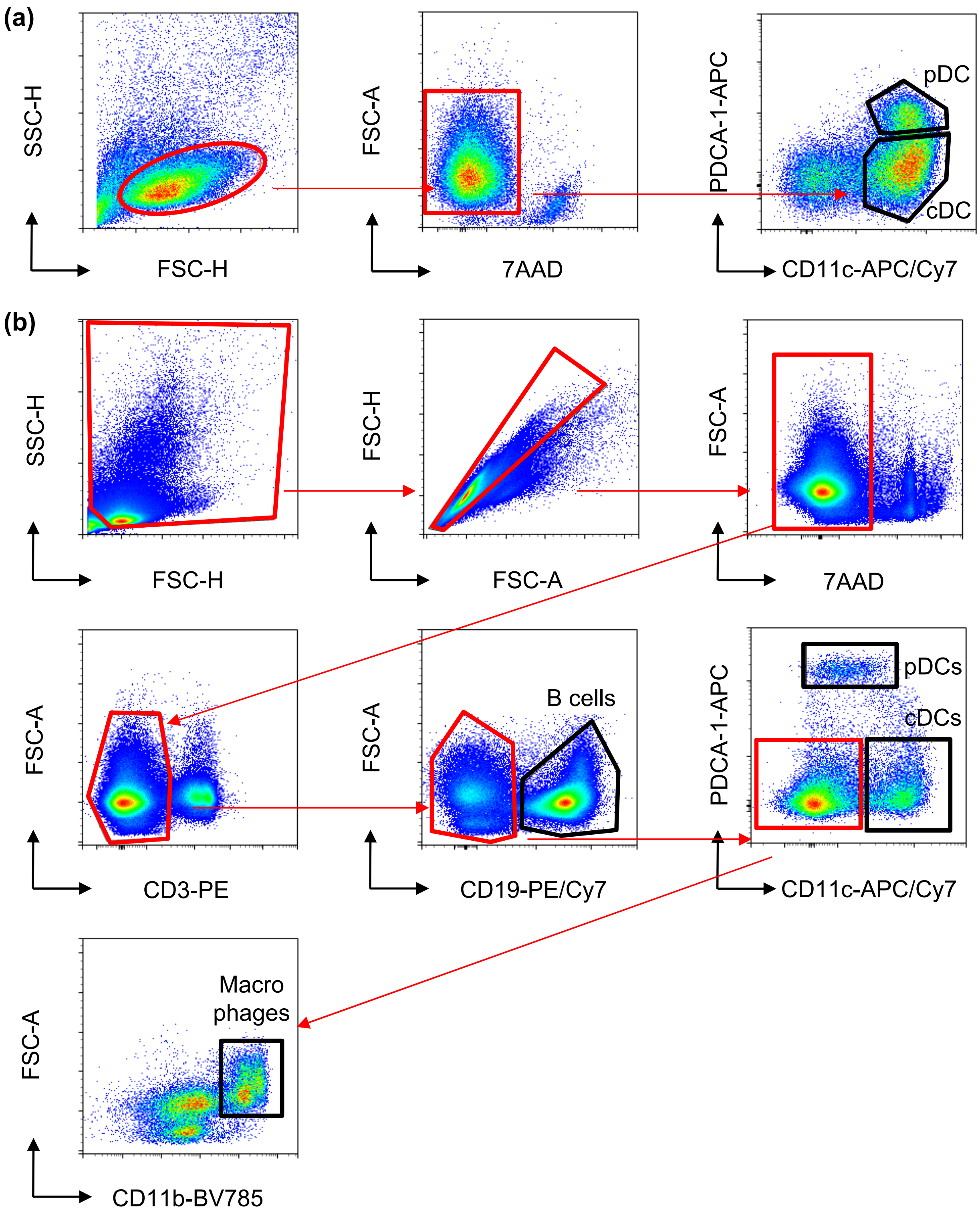

Supplementary Fig. 1: Gating strategy for flow cytometric identification of subpopulations. (a) Murine BMDCs separated into CD11c$^+$ PDCA-1$^-$ cDCs and CD11c$^+$ PDCA-1$^+$ pDCs. (b) B cells defined as CD3$^-$ CD19$^+$, cDCs as CD3$^-$ CD19$^-$ CD11c$^+$ PDCA-1$^-$, pDCs as CD3$^-$ CD19$^-$ CD11c$^+$ PDCA-1$^+$, and macrophages as CD3$^-$ CD19$^-$ CD11c$^-$ PDCA-1$^-$ CD11b$^+$.

Supplementary Figure 1

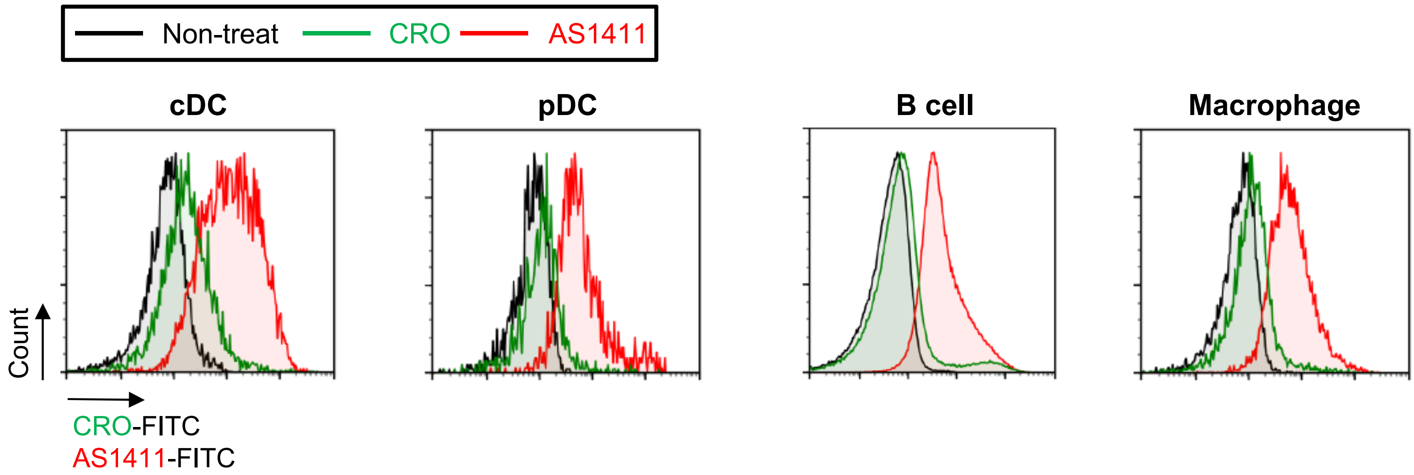

Supplementary Fig. 2: Nucleolin expression on DCs, B cells, and macrophages from mouse splenocytes. Cells were treated with 1 μM FITC-labeled AS1411 or 1 μM FITC-labeled CRO at 4 °C for 1 h and analyzed by flow cytometry. Splenocytes were separated into $CD11c^{+}$ $PDCA\text{-}1^{-}$ cDCs, $CD11c^{+}$ $PDCA\text{-}1^{+}$ pDCs, $CD19^{+}$ B cells, and $CD11c^{-}$ $CD11b^{+}$ macrophages. Each experiment was performed more than twice.

Supplementary Figure 2

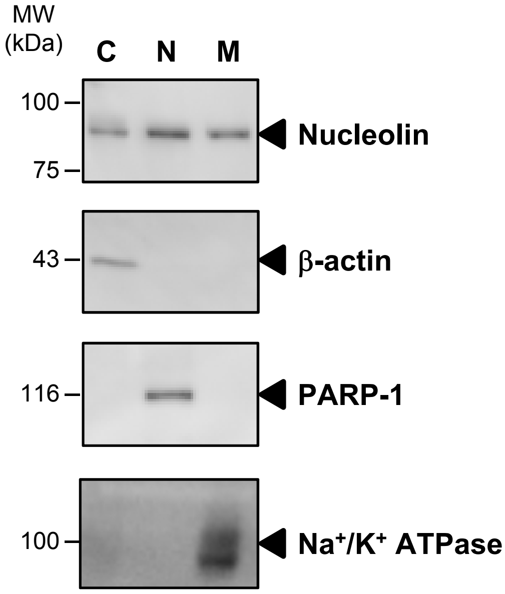

Supplementary Fig. 3: Cell-surface nucleolin on human DCs. Cytoplasmic (C), nuclear (N), and cell surface membrane (M) fractions of CAL-1 cells were collected, and localization of nucleolin was assessed by western blotting after SDS-PAGE. Fraction purity was confirmed using antibodies against β-actin for the cytoplasm fraction, PARP-1 for the nuclear fraction, and Na^{+}/K^{+}-ATPase for the cell surface membrane fraction. Each experiment was performed more than twice.

Supplementary Figure 3

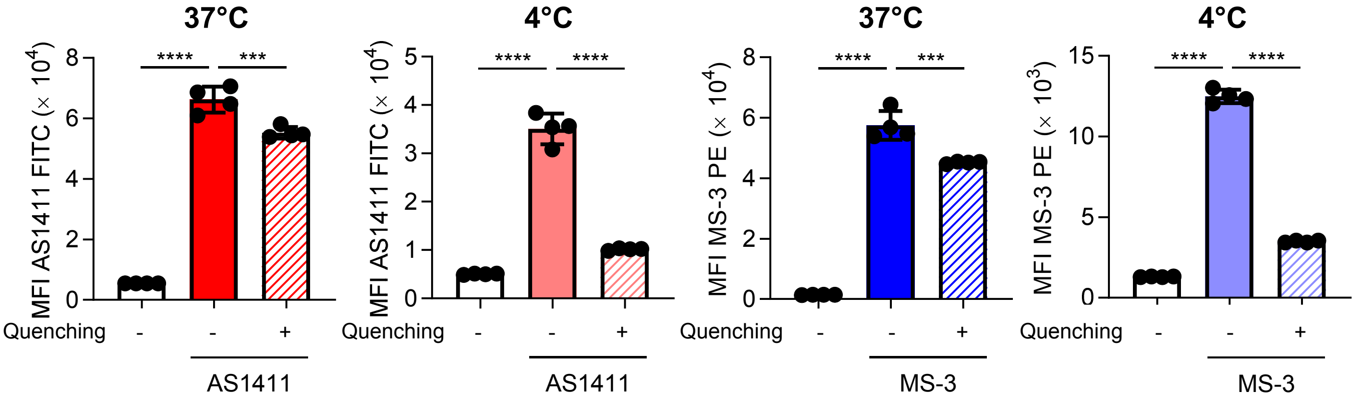

Supplementary Fig. 4: DC binding and internalization of AS1411 and MS-3. DC2.4 cells were treated with 1 μM FITC-labeled AS1411 or 10 μg/mL PE-labeled MS-3 at 37°C for 30 min or 4°C for 1 h. MFI was measured by flow cytometry. In some cases, MFI was measured by flow cytometry after trypan blue quenching. Each experiment was performed more than twice. Data are shown as means ± SD. ***P < 0.001, ****P < 0.0001 by Tukey's test.

Supplementary Figure 4

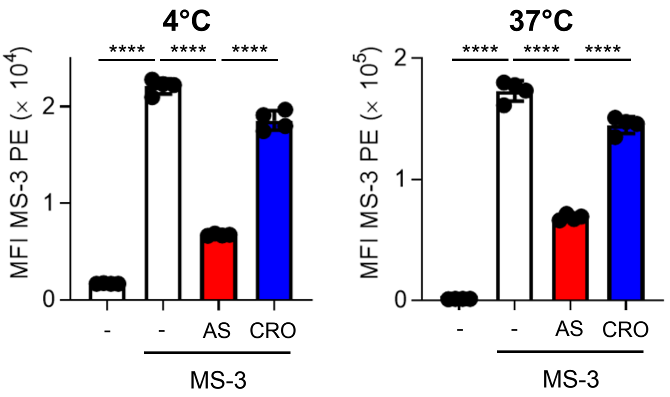

Supplementary Fig. 5: MS-3 inhibition of AS1411 binding and internalization. DC2.4 cells were treated with 10 μg/mL PE-labeled MS-3 with or without 5 μM AS1411 or 5 μM CRO at 4°C for 1 h or 37°C for 30 min. Binding and internalization were measured by flow cytometry. In the experiment, under the condition of 37°C, the MFI was measured by flow cytometry after trypan blue quenching. Each experiment was performed more than twice. Data are shown as means ± SD. ****P < 0.0001 by Tukey's test.

Supplementary Figure 5

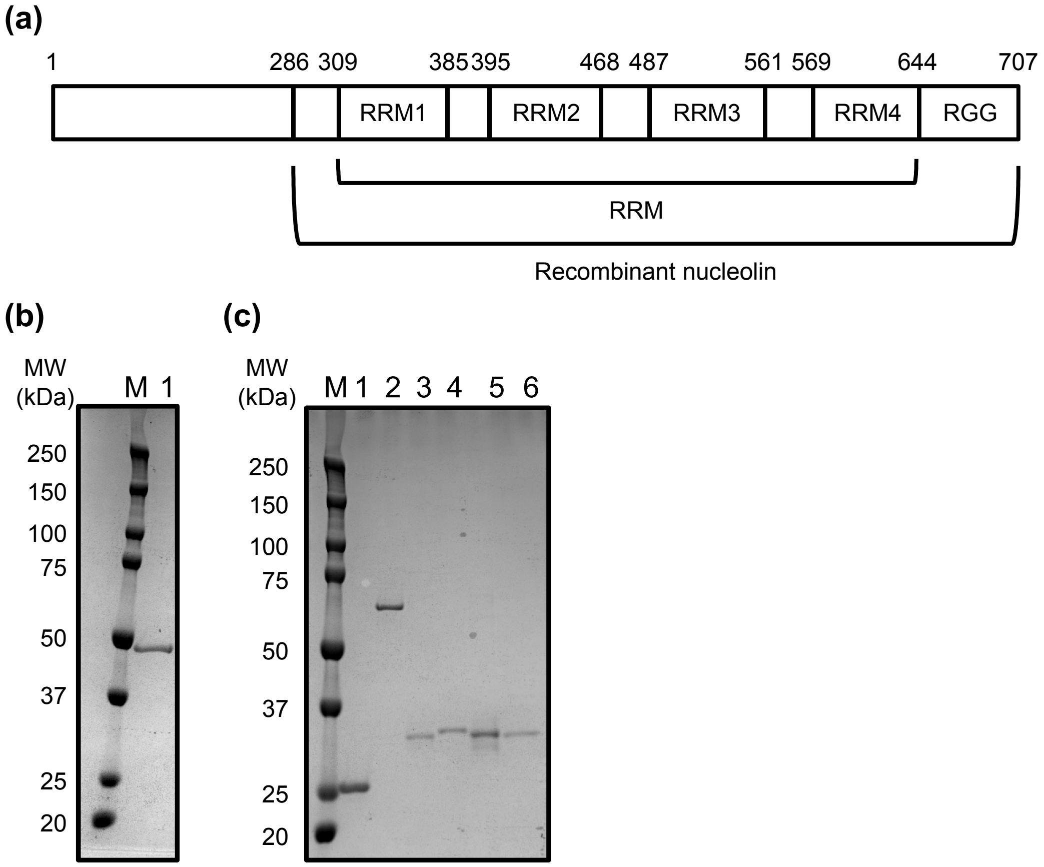

Supplementary Fig. 6: Nucleolin recombinant protein expression. (a) Nucleolin domain diagram. (b) Purified nucleolin (aa286-aa707) analyzed using SDS-PAGE followed by staining with Coomassie brilliant blue. M, marker; lane 1, nucleolin. (c) Purified RRM (aa309-aa644), RRM1 (aa309-aa385), RRM2 (aa395-aa468), RRM3 (aa487-aa561), and RRM4 (aa569-aa644) separated using SDS-PAGE followed by staining with Coomassie brilliant blue. M, marker; lane 1, GST protein; lane 2, RRM; lane 3, RRM1; lane 4, RRM2; lane 5, RRM3; lane 6, RRM4.

Supplementary Figure 6

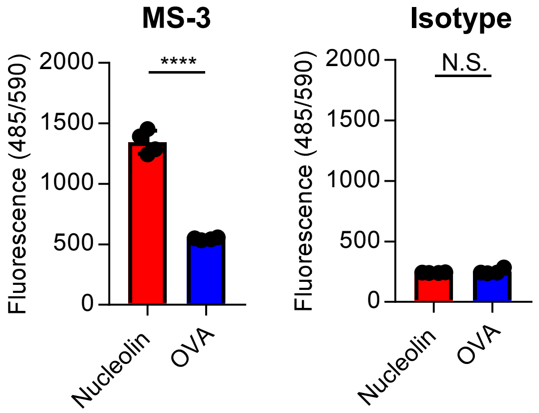

Supplementary Fig. 7: MS-3 binding to recombinant nucleolin. PE-labeled isotype control antibody and ovalbumin (OVA) are negative controls. N.S.: not significant. Each experiment was performed more than twice. Data are shown as means ± SD. ***$P < 0.001$ by Student's *t*-test.

Supplementary Figure 7

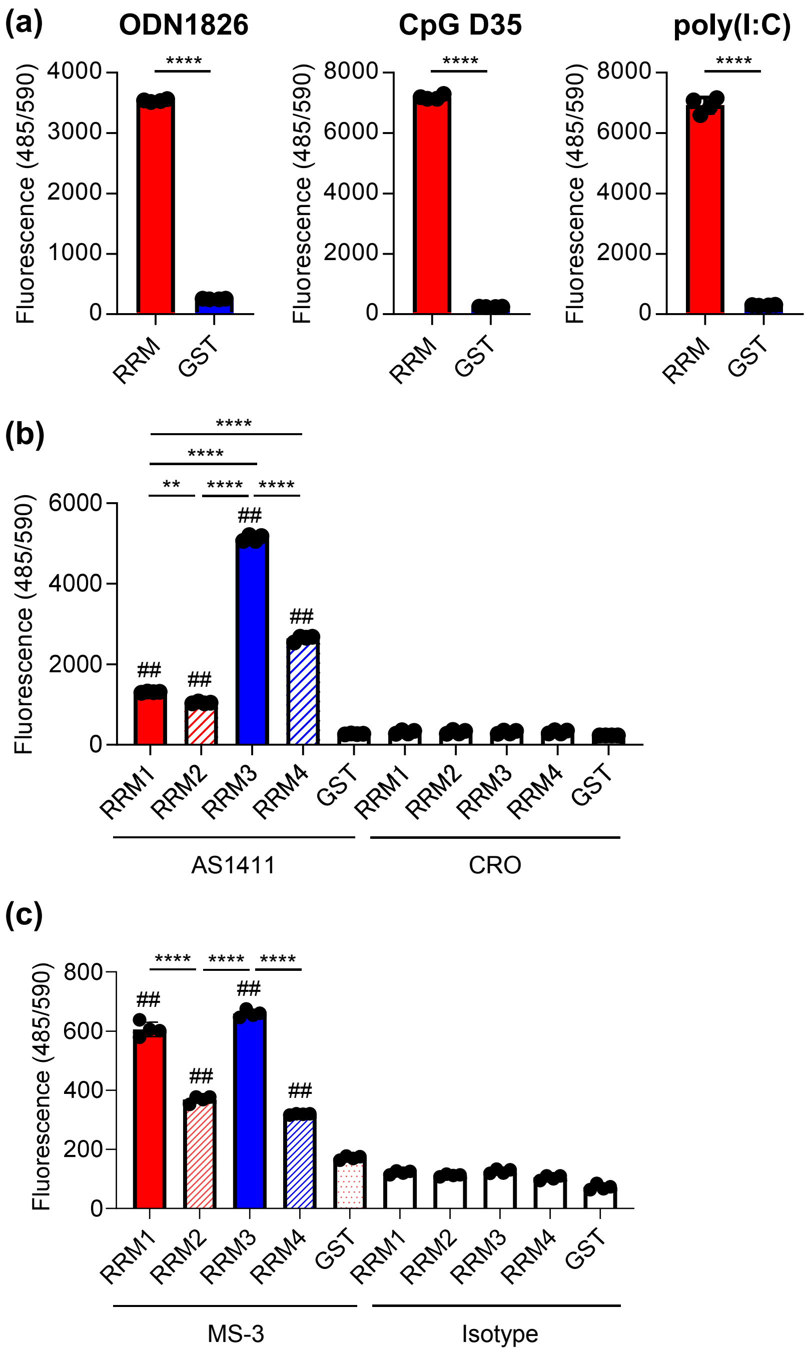

Supplementary Fig. 8: Binding of nucleic acid-based adjuvants, AS1411, and MS-3 to recombinant nucleolin proteins. (a) Binding of biotin-conjugated ODN1826, CpG D35, and poly(I:C) to recombinant RRM protein or GST protein measured using PE-labeled streptavidin. (b) Binding of biotin-conjugated AS1411 or CRO to recombinant RRM1, RRM2, RRM3, RRM4, or GST proteins measured using PE-labeled streptavidin. (c) Binding of PE-labeled MS-3 or PE-labeled isotype control antibody to recombinant RRM1, RRM2, RRM3, RRM4, or GST proteins. (b, c) N.S.: not significant. (a–c) Each experiment was performed more than twice. Data are shown as means ± SD. **$P < 0.01$, ****$P < 0.0001$ by (a) Student's *t*-test and (b, c) Tukey's test. (b, c) $^{\#\#}P < 0.0001$ vs. GST group by Tukey's test.

Supplementary Figure 8

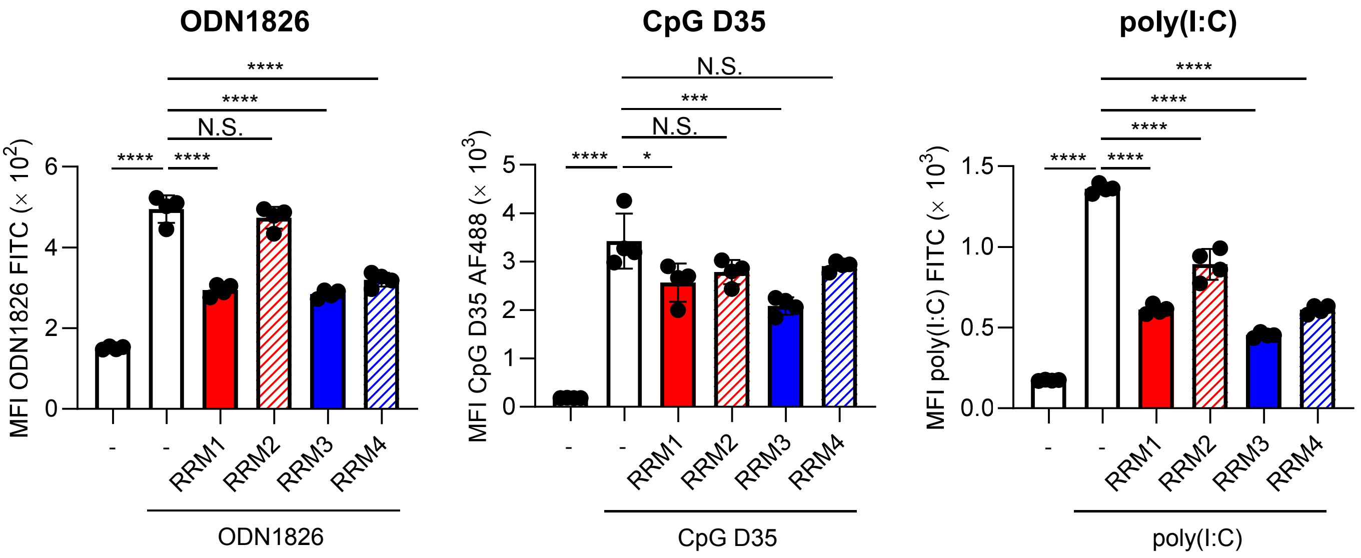

Supplementary Fig. 9: Binding of nucleic acid-based adjuvants to recombinant RRM domains. DC2.4 cells were treated with FITC-labeled ODN1826, Alexa 488-labeled CpG D35, or FITC-labeled poly(I:C), either with or without recombinant RRM1, RRM2, RRM3, or RRM4 at 37°C for 30 min. Internalization of FITC-labeled ODN1826, Alexa 488-labeled CpG D35, or FITC-labeled poly(I:C) into DC2.4 cells was measured by flow cytometry after trypan blue quenching. N.S., not significant; MFI, mean fluorescence intensity. Each experiment was performed more than twice. Data are shown as mean ± SD. *$P < 0.05$, ***$P < 0.001$, ****$P < 0.0001$ by Tukey's test.

Supplementary Figure 9

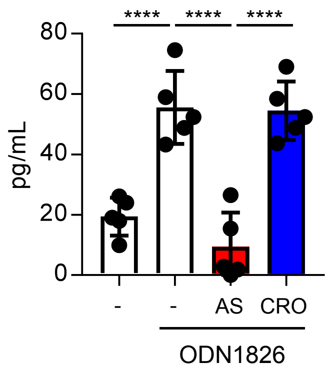

Supplementary Fig. 10: ODN1826 induces IL-12 p70 production by DCs. Murine BMDCs were treated with 0.1 μg/mL ODN1826 with or without AS1411 (5 μM) or CRO (5 μM) at 37°C for 24 h. Cytokines in supernatants were measured by ELISA. Each experiment was performed more than twice. Data are shown as means ± SD. ****P < 0.0001 by Tukey's test.

Supplementary Figure 10

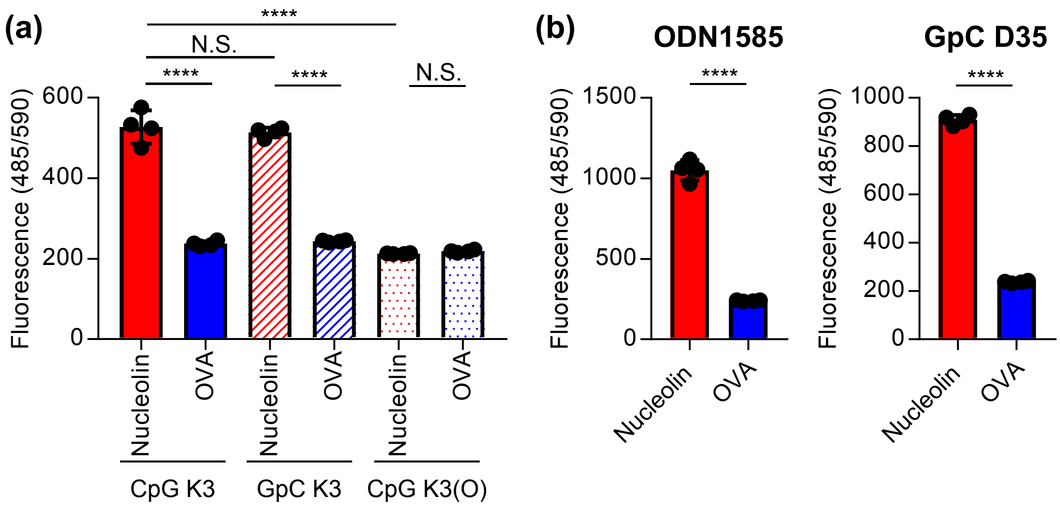

Supplementary Fig. 11: Binding of CpG ODNs to recombinant nucleolin. (a) Biotin-conjugated CpG K3, GpC K3, and CpG K3 modified with a phosphodiester backbone (CpG K3(O)) binding to nucleolin and OVA control detected using PE-labeled streptavidin. N.S.: not significant. (b) Biotin-labeled ODN1585 and GpC D35 binding to nucleolin and OVA control detected using PE-labeled streptavidin. Each experiment was performed more than twice. Data are shown as means ± SD. ****$P < 0.0001$ by (a) Tukey's test and (b) Student's *t*-test.

Supplementary Figure 11

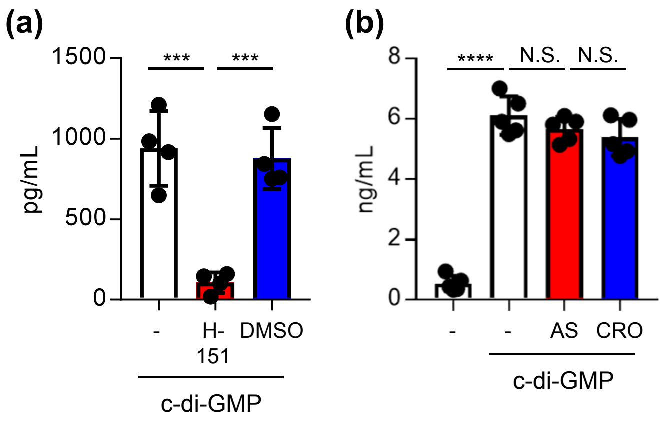

Supplementary Fig. S12: c-di-GMP treatment elicits IL-12 p40 production by murine BMDCs. Cells were treated with 10 μg/mL c-di-GMP (a) with or without 0.4 μg/mL H-151 and (b) with or without 5 μM AS1411 or 5 μM CRO at 37°C for 24 h. IL-12 p40 in supernatants was measured by ELISA. N.S.: not significant. Each experiment was performed more than twice. Data are shown as means ± SD. ***P < 0.001, ****P < 0.0001 by Tukey's test.

Supplementary Figure 12

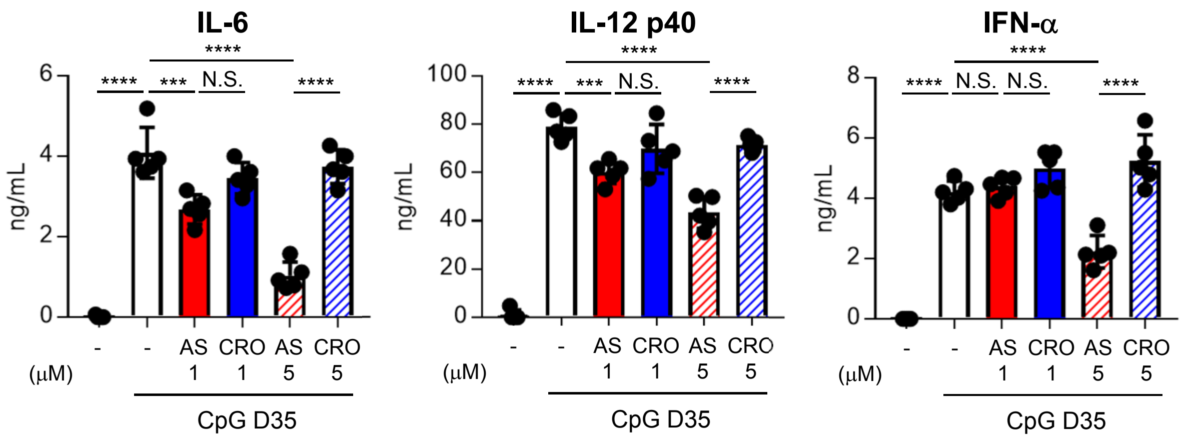

Supplementary Fig. 13: CpG D35 stimulation of cytokine production by BMDCs is nucleolin-dependent. Cells were treated with 10 μg/mL CpG D35 with or without AS1411 (1 or 5 μM) or CRO (1 or 5 μM) at 37°C for 24 h. IL-6, IL-12 p40, and IFN-α in supernatants were measured by ELISA. N.S.: not significant. Each experiment was performed more than twice. Data are shown as means ± SD. ***$P < 0.001$, ****$P < 0.0001$ by Tukey's test.

Supplementary Figure 13

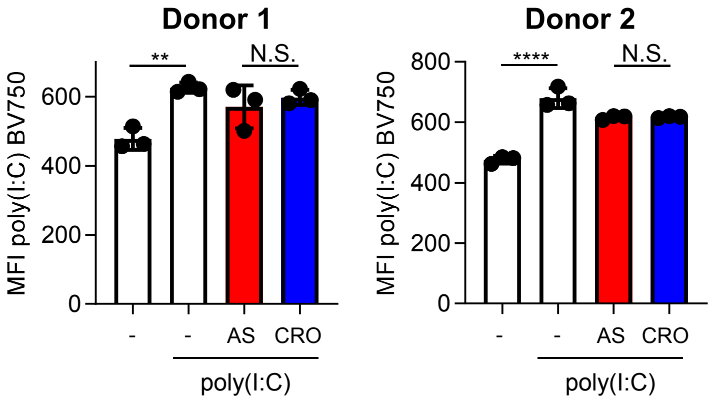

Supplementary Fig. 14. Contribution of nucleolin on poly(I:C)-induced enhanced expression of CD80 on human PBMCs. Human PBMCs were treated with 1 μg/mL poly(I:C) with or without 1 μM AS1411 or 1 μM CRO at 37°C for 12 h. The expression level of CD80 on $CD14^+$ monocytes was measured by flow cytometry. N.S.: not significant. Each experiment was performed more than twice. Data are shown as means ± SD. ****P* < 0.01, *******P* < 0.0001 by Tukey's test.

Supplementary Figure 14
