# Supplementary material for: Elucidation of the role of nucleolin as a cell surface receptor for nucleic acid-based adjuvants
Source: NPJ Vaccines. 2022 Oct 6;7:115. doi: 10.1038/s41541-022-00541-6 (PMC9537314; doi:10.1038/s41541-022-00541-6)
Supplement: Supplementary file 2 — REPORTING SUMMARY [file 41541_2022_541_MOESM2_ESM.pdf]

nature portfolio

Corresponding author(s): Dr Yasuo Yoshioka

Last updated by author(s): Sep 8, 2022

Reporting Summary

Nature Portfolio wishes to improve the reproducibility of the work that we publish. This form provides structure for consistency and transparency in reporting. For further information on Nature Portfolio policies, see our Editorial Policies and the Editorial Policy Checklist.

Statistics

For all statistical analyses, confirm that the following items are present in the figure legend, table legend, main text, or Methods section.

n/a	Confirmed	
☐	☒	The exact sample size (*n*) for each experimental group/condition, given as a discrete number and unit of measurement
☐	☒	A statement on whether measurements were taken from distinct samples or whether the same sample was measured repeatedly
☐	☒	The statistical test(s) used AND whether they are one- or two-sided<br>*Only common tests should be described solely by name; describe more complex techniques in the Methods section.*
☒	☐	A description of all covariates tested
☒	☐	A description of any assumptions or corrections, such as tests of normality and adjustment for multiple comparisons
☐	☒	A full description of the statistical parameters including central tendency (e.g. means) or other basic estimates (e.g. regression coefficient) AND variation (e.g. standard deviation) or associated estimates of uncertainty (e.g. confidence intervals)
☒	☐	For null hypothesis testing, the test statistic (e.g. *F*, *t*, *r*) with confidence intervals, effect sizes, degrees of freedom and *P* value noted<br>*Give P values as exact values whenever suitable.*
☒	☐	For Bayesian analysis, information on the choice of priors and Markov chain Monte Carlo settings
☒	☐	For hierarchical and complex designs, identification of the appropriate level for tests and full reporting of outcomes
☒	☐	Estimates of effect sizes (e.g. Cohen's *d*, Pearson's *r*), indicating how they were calculated

Our web collection on statistics for biologists contains articles on many of the points above.

Software and code

Policy information about availability of computer code

Data collection	Western blotting was detected using a ChemiDoc Touch Imaging System (Bio-Rad).<br>Fluorescence was measured using a PowerScanHT (DS Pharma Biomedical).<br>ELISA plates were read using a Power Wave HT (BioTek).
Data analysis	GraphPad Prism version 7, FlowJo version 10

For manuscripts utilizing custom algorithms or software that are central to the research but not yet described in published literature, software must be made available to editors and reviewers. We strongly encourage code deposition in a community repository (e.g. GitHub). See the Nature Portfolio guidelines for submitting code & software for further information.

Data

Policy information about availability of data

All manuscripts must include a data availability statement. This statement should provide the following information, where applicable:

- Accession codes, unique identifiers, or web links for publicly available datasets
- A description of any restrictions on data availability
- For clinical datasets or third party data, please ensure that the statement adheres to our policy

The data supporting the findings of this study are presented in the article and supplementary material. Further information and requests for resources and reagents should be directed to and fulfilled by the lead contact Yasuo Yoshioka (y-yoshioka@biken.osaka-u.ac.jp).

Human research participants

Policy information about studies involving human research participants and Sex and Gender in Research.

Reporting on sex and gender	We collected data on sex, self-reported.
Population characteristics	Human PBMCs were obtained from two healthy adult male and female Japanese volunteers who provided informed consent.
Recruitment	We recruited healthy and non-pregnant volunteers aged 22–45 years using the university's email list.
Ethics oversight	All experiments using human PBMCs were approved by the Institutional Review Board of the Research Institute for Microbial Diseases, Osaka University (protocol number 31-3-1) and were followed in compliance with the Declaration of Helsinki 1975; all participants provided written informed consent.

Note that full information on the approval of the study protocol must also be provided in the manuscript.

Field-specific reporting

Please select the one below that is the best fit for your research. If you are not sure, read the appropriate sections before making your selection.

[x] Life sciences [ ] Behavioural & social sciences [ ] Ecological, evolutionary & environmental sciences

For a reference copy of the document with all sections, see nature.com/documents/nr-reporting-summary-flat.pdf

Life sciences study design

All studies must disclose on these points even when the disclosure is negative.

Sample size	The sample size were chosen based on the previous experience that can be analyzed for the statistical significance.
Data exclusions	No experiment data were excluded from the analyses.
Replication	All experiments were performed more than twice to ensure reproducibility.
Randomization	All animals were randomly assigned to treatment.
Blinding	We were not blinded to group allocation during experiments.

Reporting for specific materials, systems and methods

We require information from authors about some types of materials, experimental systems and methods used in many studies. Here, indicate whether each material, system or method listed is relevant to your study. If you are not sure if a list item applies to your research, read the appropriate section before selecting a response.

Materials & experimental systems

n/a	Involved in the study	
[ ]	[x]	Antibodies
[ ]	[x]	Eukaryotic cell lines
[x]	[ ]	Palaeontology and archaeology
[ ]	[x]	Animals and other organisms
[x]	[ ]	Clinical data
[x]	[ ]	Dual use research of concern

Methods

n/a	Involved in the study	
[x]	[ ]	ChIP-seq
[ ]	[x]	Flow cytometry
[x]	[ ]	MRI-based neuroimaging

Antibodies

Antibodies used	Merck Millipore<br>Goat polyclonal anti-mouse IgG with HRP (Cat# AP503P)<br><br>SouthernBiotech<br>Goat polyclonal anti-mouse IgG1 with HRP (Cat# 1070-05)<br>Goat polyclonal anti-mouse IgG2c with HRP (Cat# 1079-05)

BioLegend
Anti-mouse CD16/CD32 (Cat# 101302, clone: 93)
Alexa Fluor 700 anti-mouse CD19 (Cat# 115528, clone: 6D5)
APC anti-mouse PDCA1 (Cat# 127016, clone: 927)
PerCP/Cy5.5 anti-mouse CD11c (Cat# 117328, clone: N418)
APC/Cy7 anti-mouse CD11c (Cat# 117324, clone: N418)
PE anti-mouse CD86 (Cat# 105008, clone: GL-1)
PE/Cy7 anti-mouse CD11b (Cat# 101216, clone: M1/70)
PE anti-mouse CD3ε (Cat# 103008, clone: 145-2C11)
BV785 anti-mouse/human CD11b (Cat# 101243, clone: M1/70)
PE Mouse IgG1, κ Isotype Ctrl antibody (Cat# 400112, clone: MOPC-21)

Invitrogen
PE-Texas Red anti-human HLA-DR (Cat# MHLDR17, clone: TU36)

BD Biosciences
APC/Cy7 anti-human CD3 (Cat# 557757, clone: SP34-2)
BUV805 anti-human CD14 (Cat# 565779, clone: M5E2)
Alexa Fluor 647 anti-human CD16 (Cat# 557710, clone: 3G8)
BUV661 anti-human CD19 (Cat# 750536, clone: SJ25C1)
Alexa Fluor 700 anti-human CD56 (Cat# 561902, clone: B159)
BV750 anti-human CD80 (Cat# 747001, clone: L307.4)

Santa Cruz
C23 antibody (Cat# sc-8031, clone: MS-3)
C23 PE antibody (Cat# sc-8031 PE, clone: MS-3)
C23 antibody (Cat# sc-17826, clone: D-6)
PARP-1 antibody (Cat# sc-8007, clone: F-2)

Sigma-Aldrich
Monoclonal Anti-β-Actin antibody produced in mouse (Cat# A2228)

Abcam
Anti-Sodium Potassium ATPase antibody - Plasma Membrane Loading Control (Cat# ab76020, clone: EP1845Y)

InvivoGen
Mouse Control IgG1 (Cat# mabg1-ctrlm, clone: T8E5)

MBL
Anti-IgG (H+L chain) (Rabbit) pAb-HRP (Cat# 458)

Validation

There are validated by the manufacturer.

Eukaryotic cell lines

Policy information about cell lines and Sex and Gender in Research

Cell line source(s)

DC2.4 cells was provided by Dr. KL Rock (Department of Pathology, University of Massachusetts Medical School, Worcester, MA, USA). CAL-1 cells was provided by Dr. Takahiro Maeda (Nagasaki University, Nagasaki, Japan).

Authentication

The cell identity was verified by the providers.

Mycoplasma contamination

We confirmed that all cell lines were negative for mycoplasma contamination.

Commonly misidentified lines
(See ICLAC register)

N/A

Animals and other research organisms

Policy information about studies involving animals; ARRIVE guidelines recommended for reporting animal research, and Sex and Gender in Research

Laboratory animals

Female C57BL/6J mice (6–7-week-old) were used.

Wild animals

N/A

Reporting on sex

Female

Field-collected samples

N/A

Ethics oversight

Animal experiments were conducted according to Osaka University's institutional guidelines for the ethical treatment of animals and

Ethics oversight	were approved by the Animal Care and Use Committee of the Research Institute for Microbial Diseases, Osaka University, Japan (protocol number, BIKEN-AP-R01-15-1).

Note that full information on the approval of the study protocol must also be provided in the manuscript.

Flow Cytometry

Plots

Confirm that:

☒ The axis labels state the marker and fluorochrome used (e.g. CD4-FITC).

☒ The axis scales are clearly visible. Include numbers along axes only for bottom left plot of group (a 'group' is an analysis of identical markers).

☒ All plots are contour plots with outliers or pseudocolor plots.

☒ A numerical value for number of cells or percentage (with statistics) is provided.

Methodology

Sample preparation	To generate murine BMDCs, we isolated bone marrow cells from the femurs of C57BL/6J mice and cultured them at 37°C for 7 or 9 days with 100 or 300 ng/mL human Fms-related tyrosine kinase 3 ligand.<br><br>Human PBMCs were isolated from whole human blood by centrifugation with Ficoll-Paque at 1,500 rpm for 15 min at 24°C. PBMCs were cultured in RPMI1640 medium supplemented with 10% fetal calf serum and 1% penicillin and streptomycin.<br><br>Cells were stained as described in the methods section.
Instrument	NovoCyte Flow Cytometer, FACSymphony A5
Software	FlowJo version 10.7.1 and 10.8
Cell population abundance	Cell sorting was not used in the study.
Gating strategy	Gating strategy was described in the methods section and shown in the Supplementary Figure.

☒ Tick this box to confirm that a figure exemplifying the gating strategy is provided in the Supplementary Information.
